# Microglia prevent peripheral immune cell invasion and promote an anti-inflammatory environment in the brain of APP-PS1 transgenic mice

**DOI:** 10.1186/s12974-018-1304-4

**Published:** 2018-09-21

**Authors:** M. S. Unger, P. Schernthaner, J. Marschallinger, H. Mrowetz, L. Aigner

**Affiliations:** 1 Institute of Molecular Regenerative Medicine, Paracelsus Medical University, Strubergasse 21, 5020 Salzburg, Austria; 2 Spinal Cord Injury and Tissue Regeneration Center Salzburg (SCI-TReCS), Paracelsus Medical University, Salzburg, Austria; 3 Department of Neurology and Neurological Sciences, Stanford University School of Medicine, Stanford, USA

**Keywords:** Alzheimer’s disease, Microglia, TMEM119, Macrophages, T-cells

## Abstract

**Background:**

Undoubtedly, neuroinflammation is a major contributor to Alzheimer’s disease (AD) progression. Neuroinflammation is characterized by the activity of brain resident glial cells, in particular microglia, but also by peripheral immune cells, which infiltrate the brain at certain stages of disease progression. The specific role of microglia in shaping AD pathology is still controversially discussed. Moreover, a possible role of microglia in the interaction and recruitment of peripheral immune cells has so far been completely ignored.

**Methods:**

We ablated microglia cells in 12-month-old WT and APP-PS1 transgenic mice for 4 weeks using the CSF1R inhibitor PLX5622 and analyzed its consequences to AD pathology and in particular to peripheral immune cell infiltration.

**Results:**

PLX5622 treatment successfully reduced microglia numbers. Interestingly, it uncovered a treatment-resistant macrophage population (Iba1^+^/TMEM119^−^). These cells strongly expressed the phagocytosis marker CD68 and the lymphocyte activation, homing, and adhesion molecule CD44, specifically at sites of amyloid-beta plaques in the brains of APP-PS1 mice. In consequence, ablation of microglia significantly raised the number of CD3^+^/CD8^+^ T-cells and reduced the expression of anti-inflammatory genes in the brains of APP-PS1 mice.

**Conclusion:**

We conclude that in neurodegenerative conditions, chronically activated microglia might limit CD3^+^/CD8^+^ T-cell recruitment to the brain and that local macrophages connect innate with adaptive immune responses. Investigating the role of peripheral immune cells, their interaction with microglia, and understanding the link between innate and adaptive immune responses in the brain might be a future directive in treating AD pathology.

**Electronic supplementary material:**

The online version of this article (10.1186/s12974-018-1304-4) contains supplementary material, which is available to authorized users.

## Background

Alzheimer’s disease (AD) is an age-related human neurodegenerative disease with a complex pathology leading to a progressive and detrimental cognitive decline (reviewed in [1]). Among the major histopathological hallmarks, i.e. amyloid-beta plaque and neurofibrillary tangle formation (reviewed in [2–6]), neuroinflammation is described as an important contributor to AD pathology (reviewed in [7–9]). Microglia, the brains resident immune cells, are a key element in inflammatory processes of the central nervous system (CNS) and are mediating chronic neuroinflammation and aggravation of AD pathology (reviewed in [8, 10, 11]). Indeed, genome-wide association studies linked microglia (dys-) functions to AD [12, 13], and therefore, modulation of microglia phenotypes and functions is a promising target for possible treatment options (reviewed in [10, 14]). Besides the creation of a disease-stage-specific pro- or anti-inflammatory environment, one of the main functions of microglia is to phagocytose and degrade dying cells, cellular debris, and toxic molecules (reviewed in [15]), as for example amyloid-beta along AD pathology (reviewed in [16, 17] and [18]). While the initial immune response to amyloid-beta is described as beneficial because it counteracts plaque formation [19], chronically activated microglia stir disease progression through the secretion of pro-inflammatory cytokines and neurotoxic factors (reviewed in [20]). Ultimately, microglia cells might become dysfunctional along brain aging and in neurodegenerative conditions and switch their phenotype into a senescent state with impaired phagocytosis (reviewed in [21]). In summary, microglia have multiple and extremely disease-stage-specific roles and functions in AD pathology.

Besides the brain’s innate immune system, i.e. the resident microglia, peripheral macrophages (reviewed in [22, 23]) as well as cells from the adaptive immune system are increasingly recognized as being involved in AD pathology [24–26]. Macrophages, i.e. bone marrow-derived monocytes, infiltrate the brains of transgenic AD mice [27] and participate in amyloid clearance (reviewed in [28–30]). Moreover, T-cell lymphocyte populations infiltrate the brains of transgenic AD mice in high numbers [25, 31] and were detected in human AD post-mortem brains [32–34]. The exact function of lymphocyte subsets and their contribution to AD pathology is completely unknown ([24, 25, 36] and reviewed in [35]). We recently identified a CD45^+^/CD8^+^ T-cell lymphocyte population present in the brains of transgenic APP-PS1 mice that are placed in close proximity to microglia suggesting that microglia might be involved in the recruitment of T-cells to the brain and that these two cell populations might interact and influence each other [37]. In order to experimentally address these hypotheses, we ablated microglia cells from 12-month-old WT and APP-PS1 transgenic mice for 4 weeks using the colony stimulating factor 1 receptor (CSF1R) inhibitor PLX5622 and analyzed its consequences on behavior, on amyloid plaque pathology, on the recruitment of peripheral immune cells from the innate and adaptive immune system, i.e. macrophages and T-cells, and on the expression of typical pro-inflammatory, anti-inflammatory, and phagocytosis specific-genes.

## Methods

### Compounds

PLX5622 was provided by Plexxikon Inc. and formulated in AIN-76A standard chow by Research Diets Inc. at 1200 mg/kg, as previously described [38, 39].

### Animals

Female and male APP Swedish PS1 dE9 mice (reviewed in [40, 41]) expressing a chimeric mouse/human mutant amyloid precursor protein (Mo/HuAPP695swe) and a mutant human presenilin 1 (PS1-dE9) both directed to CNS neurons under the prion protein promoter (available by Jackson Laboratory, http://www.jax.org/strain/005864) were used. Mice were housed at the Paracelsus Medical University Salzburg in groups under standard conditions at a temperature of 22 °C and a 12-h light/dark cycle with ad libitum access to standard food and water. Animal care, handling, genotyping, and experiments were approved by local ethical committees (BMWFW-66.019/0032-WF/V/3b/2016).

**Fig. 1:**
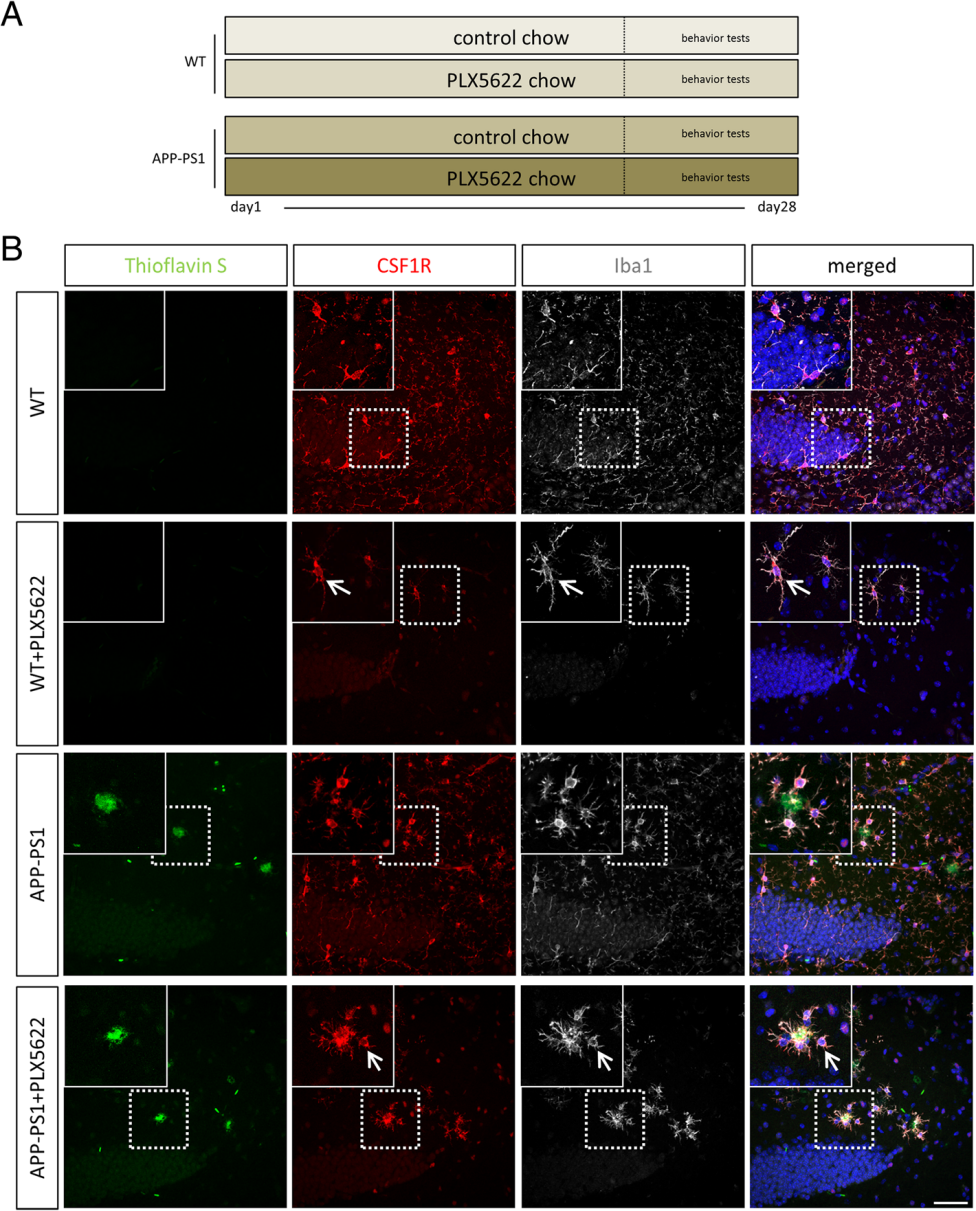
Experimental set up and expression of CSF1R in mouse hippocampus: 12-month-old WT and APP-PS1 animals were treated for a total of 28 days with PLX5622 or control chow followed by behavior tests after 20 days of treatment (**a**). Qualitative immunohistochemical staining for CSF1R receptor (red) showed high-receptor expression in Iba1^+^ cells (white) in all studied groups (**b**). ThioflavinS was used to stain amyloid plaques (green), and Dapi (blue) was used as nucleus stain. Scale: 50 μm

For this study, 12-month old animals were used and treated for 28 days with PLX5622 chow. Age-matched non-transgenic mice, derived from the breeding of APP Swedish PS1 dE9 (herein abbreviated as APP-PS1) were used as control animals (WT). All animals were adapted to control chow 2 weeks before introducing the PLX5622 chow. Thus, there were 4 experiment groups: WT and APP-PS1 mice which received control chow, and WT and APP-PS1 mice which received the PLX5622 chow for a total of 28 days (see Fig. 1a).

### Behavioral tests

All behavior tests were performed in a special animal experiment room at the animal facility of the Paracelsus Medical University under constant light and environmental conditions. After 21 days of PLX5622 treatment, behavioral tests were performed as previously published [42]. All behavior tests were conducted at the same day time and camera tracked using EthoVision tracking system (EthoVision XT 9.0.726, Noldus).

#### Morris water maze

Morris water maze (MWM) is a standard cognitive behavior test for spatial learning and memory function [43, 44]. MWM was performed on six consecutive days, starting on day 23 of the PLX5622 treatment. The maze consisted of a 108-cm round white pool that was filled with 22 °C warm water up to 1 cm above a transparent plastic platform that was placed in the southwest quadrant of the arena. The 10 × 10 cm platform was hidden under the water surface and was visually not detectable for the animals. Every mouse was put in the maze for 60 s, and the activity (e.g. latency to platform, swim speed, distance moved) until the mouse reached the platform was recorded. If the mouse does not reach the platform after 60 s, it was manually cued to the platform for orientation before it was taken out of the maze. Each mouse had to perform four trials per day for five consecutive days. Every trial per day started at four different visually marked entry points (square, triangle, circle, moon), and the entry points to the maze were randomly shuffled over the 5 days to avoid any learning effects caused by memory of the entry point. After each trial, the animals had 1 h to rest before starting a new trial. On the first day, the platform was emphasized with a flag to help the mice to find the platform and the data from day 1 were therefore excluded from the analysis. On the sixth day, the mice were additionally tested for spatial memory by removing the platform. The mice had 60 s to search the original spatial location of the platform.

### Perfusion and tissue sectioning

After 28 days of treatment, the mice were anesthetized by intraperitoneal injection of a ketamine (20.5 mg/ml, Richter Pharma), xylazine (5.36 mg/ml, Chanelle), and acepromacine (0.27 mg/ml, VANA GmbH) mixture. Afterwards, their thoracic cavity was opened with an incision caudal to the sternum. Animals were manually perfused through the left ventricle with ice cold HBSS containing 15 mM HEPES (all from Thermofisher) and 0.5% glucose (Sigma) to wash out the blood. Afterwards, mice were decapitated, and brains were extracted from the skull. One total brain hemisphere was immersed in 4% paraformaldehyde (in 0.1 M sodium phosphate solution, pH = 7.4) at 4 °C for 2 days for fixation before being washed in PBS and transferred into 30% sucrose for cryoprotection. When fully soaked with sucrose, brain hemispheres were cut in 40 μm slices on dry ice using a sliding microtome (Leika) dividing one brain hemisphere in representative tenths of the brain. Sections were stored at − 20 °C in cryoprotectant solution (ethylene glycol, glycerol, 0.1 M phosphate buffer pH 7.4, 1:1:2 by volume). The other brain hemisphere was further processed for RNA extraction or flow cytometric analysis.

### Flow cytometric analysis

For analysis of microglia and T-cells, one total brain hemisphere per mouse was mechanically chopped with a razor blade and homogenized in 2 mL ice cold HBSS with 15 mM HEPES (all from Thermofisher), 0.5% glucose (Sigma), DNAse I (1:20, Worthington) and RNAsin (1:250, Promega) using a glass homogenizer. Cells were passed through a 100-μm cell strainer and rinsed two times with 2 mL ice cold HBSS (with 15 mM HEPES and 0.5% glucose). Cell suspensions were centrifuged at 340 g for 7 min at 4 °C. Myelin was removed by resuspending the cell pellet in 30% Percoll (Sigma) solution and centrifugation at 950 g for 20 min at 4 °C. Supernatant was carefully removed, and pellets containing cells of interest were diluted in HBSS followed by centrifugation at 300 g for 10 min at 4 °C. Pellets were resuspended in PBS containing fixable viability dye (1:2000, eBioscience #65–0865) for 3 min at RT and transferred to round-bottom polystyrene tubes (Corning). After centrifugation for 5 min at 300 g, cell pellets were dissolved in FACS buffer (PBS with 2% BSA and 2 mM EDTA) containing Rat Anti-Mouse CD16/CD32 Fc-Block (1:100, BD Biosciences, #553141) for 5 min at RT. Samples were centrifuged at 300 g for 5 min, and pellets dissolved in FACS buffer containing primary fluorescent-labeled antibodies. Antibody incubation was performed for 15 min at RT. Samples were washed in FACS buffer two times and centrifuged at 400 g for 5 min. Finally, cell pellets were resuspended in 500 μl FACS buffer with RNAsin (1:250, Promega #N2115) and filtered with a 30-μm cell strainer followed by flow cytometric analysis using LSR Fortessa flow cytometer (BD) with BD FACSDiva software (8.0.1, BD). The following primary antibodies were used: CD11b-PE (1:100, eBioscience #12–0112-82), CD45-PE/Cy7 (1:100, BioLegend #103114), CD3-APC (1:100, eBioscience #17–0032), CD4-eFluor450 (1:100, eBioscience #48–0041), CD8a-FITC (1:100, eBioscience #11–0081).

Single stains were performed for compensations with isolated microglia cells from APP-PS1 mouse brains for viability dye eFluor780, CD11b-PE, and CD45-PE/Cy7 and with PBMCs collected from whole mouse blood drawn from the heart for CD3-APC, CD4-eFluor450, and CD8a-FITC. As gating strategy cells of interest were taken, and cell doublets were discriminated. Single cells negative for viability dye eFluor780 were counted as living cells and gated for CD11b-PE expression for microglia analysis. CD11b-positive cell populations were further gated for CD45 expression and divided into a low (microglia) and high population (CNS/peripheral macrophages) as published by several research groups [45–48]. Total cell numbers for CD11b+, CD11b+/CD45low, and CD11b+/CD45high were measured from total brain hemispheres and calculated for 1 × 10^6^ living cells in each sample. For T-cell analysis in the brain, single-living cells were gated for CD3-APC and CD45-PE/Cy7 expression and further analyzed for CD4-eFluor450 and CD8-FITC expression. Total cell numbers for CD3+, CD3+/CD4+, and CD3+/CD8+ were measured and calculated for 1 × 10^5^ living cells in each sample. Flow cytometric data analysis and graphs were done with Kaluza analysis software (1.3 Beckman Coulter).

### Fluorescence immunohistochemistry (IHC)

Fluorescence immunohistochemistry of mouse tissue was performed on free-floating sections as previously described [49, 50]. Antigen retrieval was performed depending on the used primary antibodies by steaming the sections for 15–20 min in citrate buffer (pH = 6.0, Sigma). The following primary antibodies were used: rabbit or goat anti-Iba1 (1:1000 or 1:500, Abcam), rabbit anti-Iba1 (1:300, Wako), rabbit anti-CSF1R (1:200, Cell Signaling), rabbit anti-TMEM119 (1:300, Abcam) and rat anti-CD44 (1:500, BioLegend), rat anti-CD8 (1:100, eBioscience), rabbit anit-Zap70 (1:400, Cell Signaling), rat anti-MHCII (1:100, eBioscience), rat anti-CD68 (1:250, Serotec/Bio-Rad), rat anti-CD4 (1:100, eBioscience), and goat anti-PCNA (1:300, Santa Cruz).

Sections were extensively washed in PBS and incubated for 3 h at RT in secondary antibodies all diluted 1:1000. The following secondary antibodies were used: donkey anti-goat Alexa Fluor 488, donkey anti-rabbit Alexa Fluor 568 or Alexa Fluor 647 (all Invitrogen/Life Technologies), donkey anti-goat Alexa Fluor 647 (Jackson Immuno Research), donkey anti-rat Alexa Fluor 488, donkey anti-goat Alexa Fluor 568, goat anti-rat Alexa Fluor 568 (all Molecular Probes), and donkey anti-rat Alexa Fluor 647 (Dianova).

Nucleus counterstaining was performed with 4′,6′-diamidino-2-phenylindole dihydrochloride hydrate (DAPI 1 mg/ml, 1:2000, Sigma). For amyloid-beta plaque staining, ThioflavinS (1 mg/ml, 1:625, Sigma) was added to the secondary antibody solution. Tissue sections were additionally treated with 0.2% Sudan Black (Sigma) in 70% ethanol for 1–2 min to reduce the autofluorescence in tissues from old animals [51]. After this treatment, the sections were extensively washed in PBS and mounted onto microscope glass slides (Superfrost Plus, Thermo Scientific). Brain sections were cover slipped semi-dry in ProLong Gold Antifade Mountant (Life technologies) or Fluorescence Mounting Medium (Dako).

### Confocal microscopy and image processing

For imaging, the Confocal Laser Scanning Microscopes LSM700 and LSM710 from Zeiss were used and gratefully provided by the microscopy core facility of SCI-TReCS (Spinal Cord Injury and Tissue Regeneration Center Salzburg). Images were taken with the ZEN 2011 SP3 or SP7 (black edition) software (all from Zeiss). Quantitative analysis was done using × 20 magnification with 0.5 or 0.6 zoom, and for qualitative analysis, images were taken in × 20, × 40, or × 63 oil magnification. Images were taken as confocal z-stacks and combined to merged maximum intensity projections. For 3D reconstruction, images were processed at the LSM 710 using the Zen 2011 SP7 (black edition) software.

All images were edited and processed with the ZEN 2012 (blue edition) software (version 1.1.2.0) and Microsoft PowerPoint.

### RNA isolation and gene expression analysis

To detect mRNA levels of microglia, anti-inflammatory, pro-inflammatory, and phagocytosis-relevant genes in different brain regions of 12-month-old mice, the total RNA was extracted from mouse hippocampus and cortex. After manual perfusion, animals were decapitated and the tissue of interest was dissected of one brain hemisphere. Brain samples were immediately transferred to RNA later (Sigma) and stored at − 80 °C. Tissues were homogenized in 1 ml Trizol (TRI®Reagent; Sigma). For phase separation, 150 μl of 1-bromo-3-chloropropane (Sigma) were added, vortexed, and centrifuged (15 min at 12,000×*g* at 4 °C). After transferring the aqueous phase into a new tube, 1 μl GlycoBlue™ (Invitrogen) and 500 μl 2-Propanol p.A. (Millipore) were added and vortexed. To obtain RNA, samples were centrifuged (10 min at 12,000×*g* at 4 °C). The pellet was washed with 1 ml 75% ethanol, dried and resuspended in a 30-μl RNase-free water (pre-warmed to 55 °C). cDNA was synthesized using the iScript Reverse Transcription Supermix (Bio-Rad). Quantitative gene expression analyses were performed using TaqMan RT-PCR technology. Technical duplicates containing 10 ng of reverse transcribed RNA were amplified with the GoTAQ Probe qPCR Master Mix (Promega) using a two-step cycling protocol (95 °C for 15 s, 60 °C for 60 s; 40 cycles, Bio-Rad CFX 96 Cycler). The following validated exon-spanning gene expression assays were employed; from a set of three validated candidate housekeepers, the two best fitting were chosen for the present experiments (PSMD4, Mm.PT.56.13046188; Heatr3 Mm.PT.56.8463165; both Integrated DNA Technologies). Quantification analyses were performed with qBase Plus (Biogazelle) using geNorm algorithms for multi-reference gene normalization. Bars are represented as mean with SD (*n* = 6–8/group). Used Primer for analyzed genes: Arg1 (Mm00475988_m1), H2-Aa (Mm00439211_m1), MRC1 (Mm00485148_m1), Nos2 (Mm00440485_m1), TNF (Mm00443258_m1), and Marco (Mm00440265_m1, all from Thermofisher); CCL2 (Mm.PT.56a.42151692), IL-1ß (Mm.PT.56a.41616450), IL-6 (Mm.PT.56a.10005566), TGFß (Mm.PT.56a.11254750), INF (Mm.PT.56a.41152792), IL-10 (Mm.PT.58.13531087), CD33 (Mm.PT.58.12829132), Trem2 (Mm.PT.58.7992121), TMEM119 (Mm.PT.58.6766267), and AIF1 (Mm.PT.56a.7014816, all from Integrated DNA Technologies).

### Data analysis

#### Behavioral testing

The data of the behavioral tests were collected with EthoVision XT (version 9.0.72) software from Noldus. MWM was divided in spatial learning which consisted of the first 5 days and spatial memory analysis on the sixth day. For learning assessment, the swim speed, the total distance the animals moved, and the latency from the arena entry to the platform were calculated and plotted as learning curves with mean and standard error of the mean (SEM). To assess the spatial memory, the cumulative durations the animals spent in the platform quadrant were calculated.

#### Fluorescence immunohistochemistry (IHC)

For quantitative analysis, comparable images of the hippocampus (dentate gyrus) and the cortex from four different brain slices of each animal were taken with the LSM700 or LSM710 confocal fluorescence microscope at × 20 magnification with 0.5 or 0.6 zoom. In each image, the total number of Iba1 positive cells, Iba1^+^/ThioflavinS^+^ doublepositive cells, the number of ThioflavinS positive amyloid-beta plaques, as well as the area of the plaques were assessed. The percentage of Iba1^+^/ThioflavinS^+^positive cells from the total number of Iba1 positive cells was calculated. Furthermore, the total number of Iba1^+^/TMEM119^+^ double positive and Iba1^+^/TMEM119^−^ cells was counted. Again, the ratio of Iba1^+^/TMEM119^+^ and Iba1^+^/TMEM119^−^ cells from the total number of Iba1-positive cells was calculated in percentage (all analysis *n* = 6/group). For further characterization Iba1^+^/TMEM119^+^/CD68^+^ and Iba1^+^/TMEM119^−^/CD68^+^ cells were counted (*n* = 3/group). Only cells with visible somata and nuclei were taken for analysis.

For semi-quantitative analysis of CD44 expression, staining was performed at the same time for all groups and the percentage area of CD44 staining was calculated. Four confocal z-stacks of areas from the cortex and the hippocampus of each animal were taken (*n* = 3/group) with constant illumination and detection settings at × 20 magnification and 0.5 zoom. For every image, maximum intensity projections were generated and the threshold for the respective staining was manually set. The number of particles (bigger than 1 μm^2^) and the particle areas [square micrometers] were calculated using the ImageJ tool “Analyze particles.” The total tissue area [in square micrometers] of each image was calculated, and the area percentage (area of stained particles/field of view) was calculated. The counting and measuring procedures were done manually using ImageJ software (1.44p).

Qualitative analysis for the CSF1R staining and the CD44 staining was performed, taking images of the hippocampus and cortex from all four study groups. The images were adjusted in color, size, brightness, and contrast using the ZEN lite 2012 software (version 1.1.2.0) and Microsoft PowerPoint.

Colocalization analysis was done using Imaris Software (9.1.2, Bitplane). Four confocal images of the hippocampus or cortex per animal were taken at × 40 magnification and 0.6 zoom and processed with the “Coloc” tool of the Imaris software. Colocalization channels were created for ThioflavinS and Iba1 as well as ThioflavinS and TMEM119 after setting manually the threshold for overlapping signals. The percentage (%) of dataset colocalized for the entire z-stack was calculated in APP-PS1 and APP-PS1 PLX5622-treated animals. For analysis of specifically macrophage plaque uptake, the percentage of ThioflavinS TMEM119 colocalization was subtracted from the total percentage of ThioflavinS Iba1 colocalization dataset (% of dataset colocalized ThioflavinS^+^/Iba1^+^/TMEM119^−^; *n* = 3/group).

Quantification of CD8^+^ T-cell numbers in the total brain section was performed using a Virtual Slide Microscope VS120 with the Olympus VS-ASW.L100 software (both from Olympus). Four total sagittal brain sections of one-tenth brain hemisphere per animal (12-month-old WT and APP-PS1 mice) were scanned at × 20 magnification (*n* = 5/genotype). The total number of CD8^+^ cells was manually counted using Fiji software (ImageJ 1.51 h) and OlyVIA software (2.9, Olympus). The corresponding area (square micrometer) of the total sagittal section, the cortex, and the dorsal hippocampus was measured and multiplied by 40 to obtain the tissue volume represented in cubic micrometer (μm^3^). To assess cell densities, the total number of counted cells per animal was divided by the corresponding tissue volume and represented as cells/cubic millimeter (cells/mm^3^).

#### Statistics

For statistical analysis, the Prism 5–7 software (GraphPad) was used. The data were tested for normal distribution with the Kolmogorov-Smirnov test and were tested for outliers using Grubb’s test. Comparing two groups, the unpaired Student’s *t* test was used. Welch’s correction was performed when variances were significantly different. If more than two groups were compared, one-way analysis of variance (ANOVA) was used and for behavioral learning assessment over time, two-way ANOVA was performed. For the one-way ANOVA, the Tukey’s multiple comparison test was used as a post-hoc test, and for the two-way ANOVA, the Bonferroni or Tukey's multiple comparison post-test was performed. For gene expression data analysis two-way ANOVA with Tukey's multiple comparison was used. The data were depicted as mean and standard deviation (SD) with a 95% confidence interval or as mean with standard error of the mean (SEM) as indicated in the figure legends. *p* values of *p* < 0.0001 and *p* < 0.001 were considered extremely significant (**** or ***), *p* < 0.01 very significant (**), and *p* < 0.05 significant (*).

## Results

### CSF1R inhibition with PLX5622 diminished microglia but revealed a PLX5622-resistant population in the brains of APP-PS1 mice

To analyze the relevance of microglia in amyloid plaque pathology and CNS inflammation, we ablated these cells for a total of 28 days using the colony stimulating factor 1 receptor (CSF1R) inhibitor PLX5622 in 12-months-old APP-PS1 mice and WT littermate controls (Fig. 1a). We have chosen APP-PS1 mice with the age of 12 months, because at this stage, these animals already have massive amyloid-beta plaque formation in the hippocampus and cortex, high levels of microgliosis, and microglia activation as well as alterations in microglia cytokine production [52, 53]. The CSF1R is a cytokine receptor highly expressed on microglia and macrophages [54], and microglia survival in the adult brain strictly depends on CSF1R signaling [55]. As a consequence, inhibition of CSF1R in mice using pharmacological inhibitors such as PLX3397 leads to a total loss of about 99% of microglia cells in the brain [56]. Moreover, as a functional consequence, PLX5622-mediated ablation of microglia slightly alleviated the cognitive deficits in 3xTg-AD and 5xfAD transgenic mouse models of AD [38, 39]. Of note, peripheral macrophages and myeloid cells are less receptive to PLX5622-mediated depletion [39, 57, 58].

CSF1R is widely and exclusively expressed in Iba1^+^ cells, i.e. microglia and macrophages, of the hippocampus (Fig. 1b) and cortex (Additional file 1: Figure S1) of 12-month-old WT and APP-PS1 animals. Iba1^+^ cells co-expressed CSF1R, and in APP-PS1 mice, these cells clustered primarily at sites of amyloid plaques (Fig. 1b, APP-PS1 insert). After 28 days of treatment with PLX5622 (Fig. 1a) a few randomly distributed Iba1^+^ cells were observed in WT animals (Fig. 1b, WT + PLX5622 insert), while in PLX5622-treated APP-PS1 mice, the remaining Iba1^+^ cells were observed mainly at sites of amyloid-beta plaques (Fig. 1b, APP-PS1 + PLX5622 insert). The Iba1^+^ cells detected in the brains of PLX5622-treated WT and of APP-PS1 animals did co-express CSF1R (Fig. 1b, arrow), but seem to be resistant to the PLX5622 treatment, a fact that has been noticed also by others [39]. Similar results were observed in the cortex (Additional file 1: Figure S1).

**Fig. 2:**
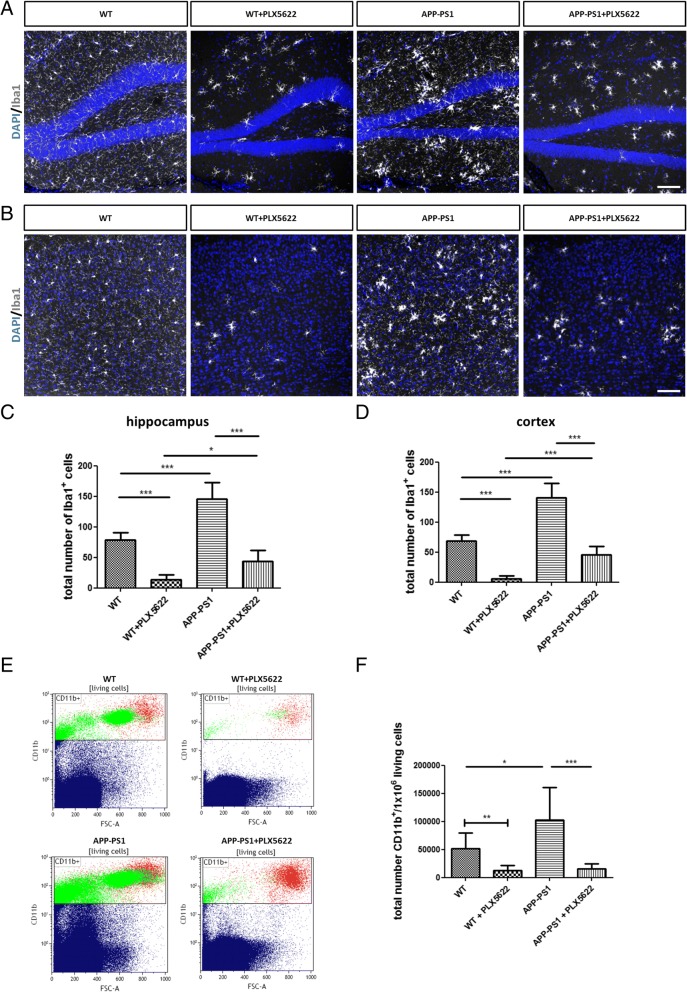
Immunohistochemical and flow cytometric analysis of microglia/macrophage cells in the brains of WT and APP-PS1 animals treated with PLX5622. Staining for Iba1 (white) in the hippocampus (**a**) and the cortex (**b**) revealed significantly reduced numbers of Iba1^+^ cells in brains of WT and APP-PS1 mice treated with PLX5622. Surprisingly, higher numbers of Iba1^+^ cells were observed in both brain regions of APP-PS1 animals before treatment and higher numbers of Iba1^+^ cells remained resistant to PLX5622 application compared to WT animals (**c**, **d**). After 28 days of treatment, microglia were mechanically isolated from total brain hemispheres, stained for CD11b, and quantitatively analyzed via flow cytometry. Representative flow cytometric dot plots of single-living CD11b^+^ cells isolated from total brain hemispheres (**e**). Both WT and APP-PS1 animals treated with PLX5622 had highly reduced numbers of CD11b^+^ cells, and surprisingly APP-PS1 animals had significantly higher numbers of isolated CD11b^+^ cells compared to WT animals (**f**). Dapi (blue) was used as nucleus stain. One-way ANOVA with Tukey’s multiple comparison test (**c**, **d** *n* = 6/group and **f** *n* = 8–9/group) and unpaired Student’s *t* test with Welch’s correction was performed comparing only WT with WT + PLX5622 (**f**, *n* = 8–9/group). Scale: 100 μm (**a**, **b**)

The 28-day PLX5622 treatment largely depleted the hippocampus and cortex of Iba1^+^ cells in both genotypes (Fig. 2a, b). Interestingly, APP-PS1 animals started out having higher numbers of Iba1^+^ cells compared to WT animals, and PLX5622-mediated Iba1 cell depletion in APP-PS1 animals was less efficient compared to WT animals. While 82.35% of Iba1 cells were ablated in WT hippocampus, only 70.04% of the cells were ablated in the APP-PS1 hippocampus (Fig. 2c). In the WT cortex, 92.12% of Iba1 cells were ablated, while in the APP-PS1 cortex, Iba1 cell ablation was 67.77% (Fig. 2d). The remaining Iba1^+^ cells in the APP-PS1 animals were especially gathered around amyloid-plaques (Fig. 1b, APP-PS1 + PLX5622). Next, we used flow cytometry analysis of all CD11b-positive cells to quantify the effects of the PLX5622 treatment on the microglia/macrophage population (Fig. 2e). First, the total number of CD11b^+^ cells isolated was significantly higher in the brains of APP-PS1 compared to WT mice (Fig. 2f). This was specifically seen in female APP-PS1 mice compared to female WT and compared to male APP-PS1 animals (Additional file 4: Figure S4, A-C). Second, after 28 days of PLX5622 treatment, the population of CD11b^+^ cells was largely diminished but not completely erased in the brains of WT and APP-PS1 animals (Fig. 2e, f). In summary, microglia/macrophage depletion by PLX5622 treatment was efficient but not complete, and it identified a PLX5622-resistant microglia/macrophage population clustering at amyloid-beta plaques in the brains of APP-PS1 mice.

### PLX5622 treatment and microglia ablation did neither modulate plaque pathology nor improve cognition in APP-PS1 mice

**Fig. 3:**
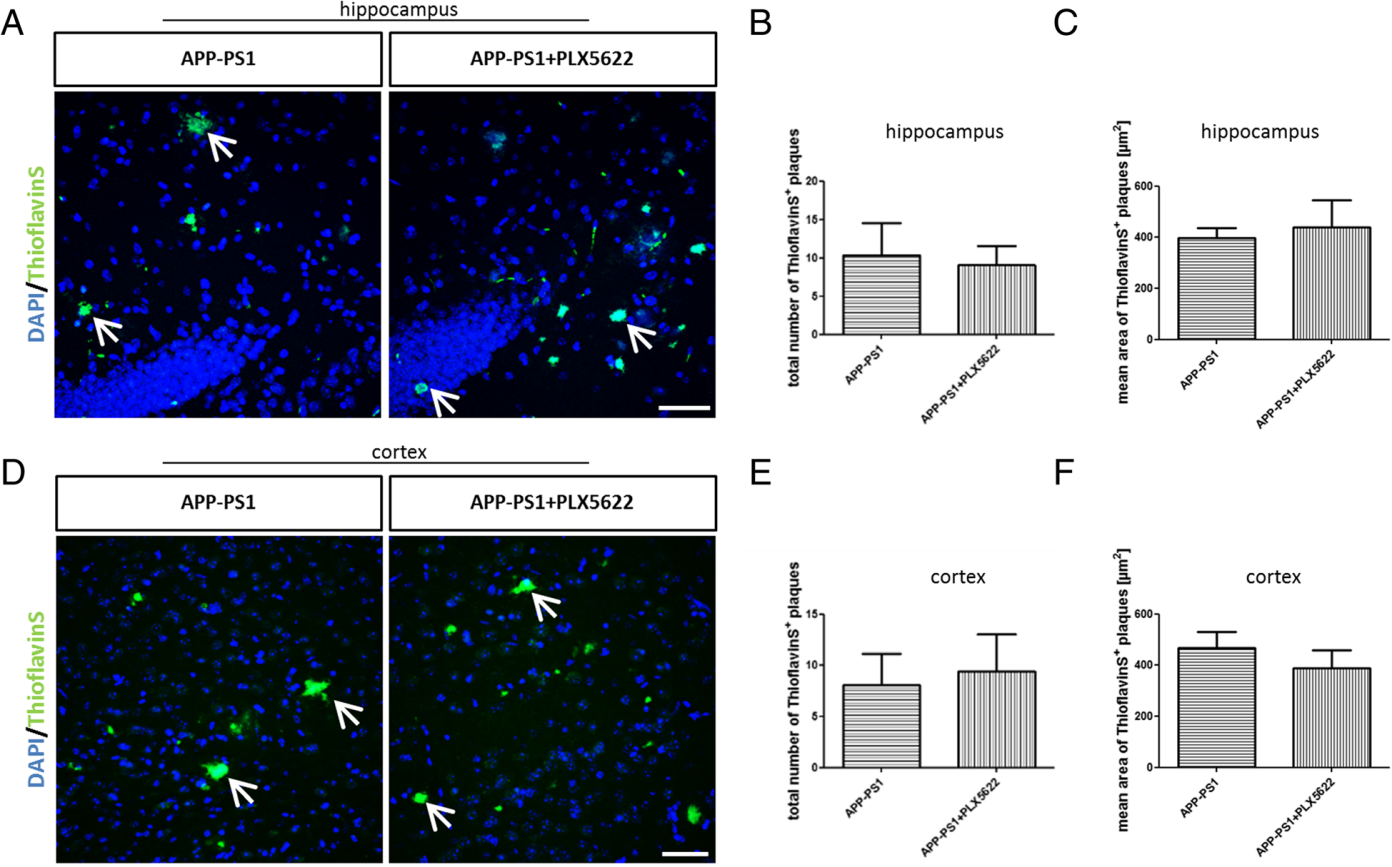
Microglia elimination via PLX5622 treatment did not change amyloid-beta plaque pathology in APP-PS1 mice. The total number of ThioflavinS (green)-positive amyloid-plaques was not changed after ablation of microglia in the hippocampus (**a**, **b**) and the cortex (**d**, **e**). Plaques did not change in size upon treatment with PLX5622 as analyzed by the mean area of ThioflavinS-positive plaques in the hippocampus (**c**) and the cortex (**f**). Dapi (blue) was used as nucleus stain. Unpaired Student’s *t* test (**b**, **e**, **f**) with Welch’s correction (**c**) was performed (*n* = 6/group). Scale: 50 μm (**a**, **d**)

Since microglia cells are highly involved in amyloid-beta clearance, we assumed that a lack of microglia might increase the plaque burden in APP-PS1 mice. Surprisingly, there was neither a change in plaque numbers nor in plaque size in the hippocampus or cortex of APP-PS1 PLX5622-treated mice (Fig. 3a–f). These data suggest that the depleted microglia might not have been a major contributor to amyloid plaque degradation at this stage of disease pathology or that the remaining PLX5622 resistant Iba1^+^ cells in APP-PS1 mice are highly efficient in amyloid phagocytosis and able to compensate the low numbers of microglia thereby keeping the plaque load at the same level. Another explanation might be that the 28-day treatment was just too short for a modulation of plaque pathology.

Next, we were interested in the consequences of the microglia depletion on cognitive function using the Morris water maze (MWM) test (Additional file 2: Figure S2). For behavior data analysis, mixed gender was used, since only minor sex differences were observed in the animals (Additional file 3: Figure S3). We analyzed the total distance the animals traveled to reach the platform from day 2 to day 5. Despite a similar swimming speed of all animals (Additional file 2: Figure S2A), APP-PS1 mice had significant learning deficits and required longer distances to reach the platform compared to WT mice (Additional file 2: Figure S2B). PLX5622 treatment had no impact on learning behavior in WT mice (Additional file 2: Figure S2C), and microglia ablation did not improve cognitive deficits in APP-PS1 animals (Additional file 2: Figure S2D, E). For analysis of spatial memory, the platform was removed on day 6 and the time the animals spent in the original platform quadrant was measured. Memory deficits were not observed in APP-PS1 compared to WT mice, and PLX5622 treatment did not alter memory function in the APP-PS1 animals. In contrast, PLX5622-treated WT mice spent less time in the platform quadrant when compared only to their respective controls (Additional file 2: Figure S2F), indicating that microglia might contribute to memory function in the healthy CNS.

### APP-PS1 brains are colonized by macrophages closely located to amyloid-beta plaques and are less susceptible to PLX5622 treatment

**Fig. 4:**
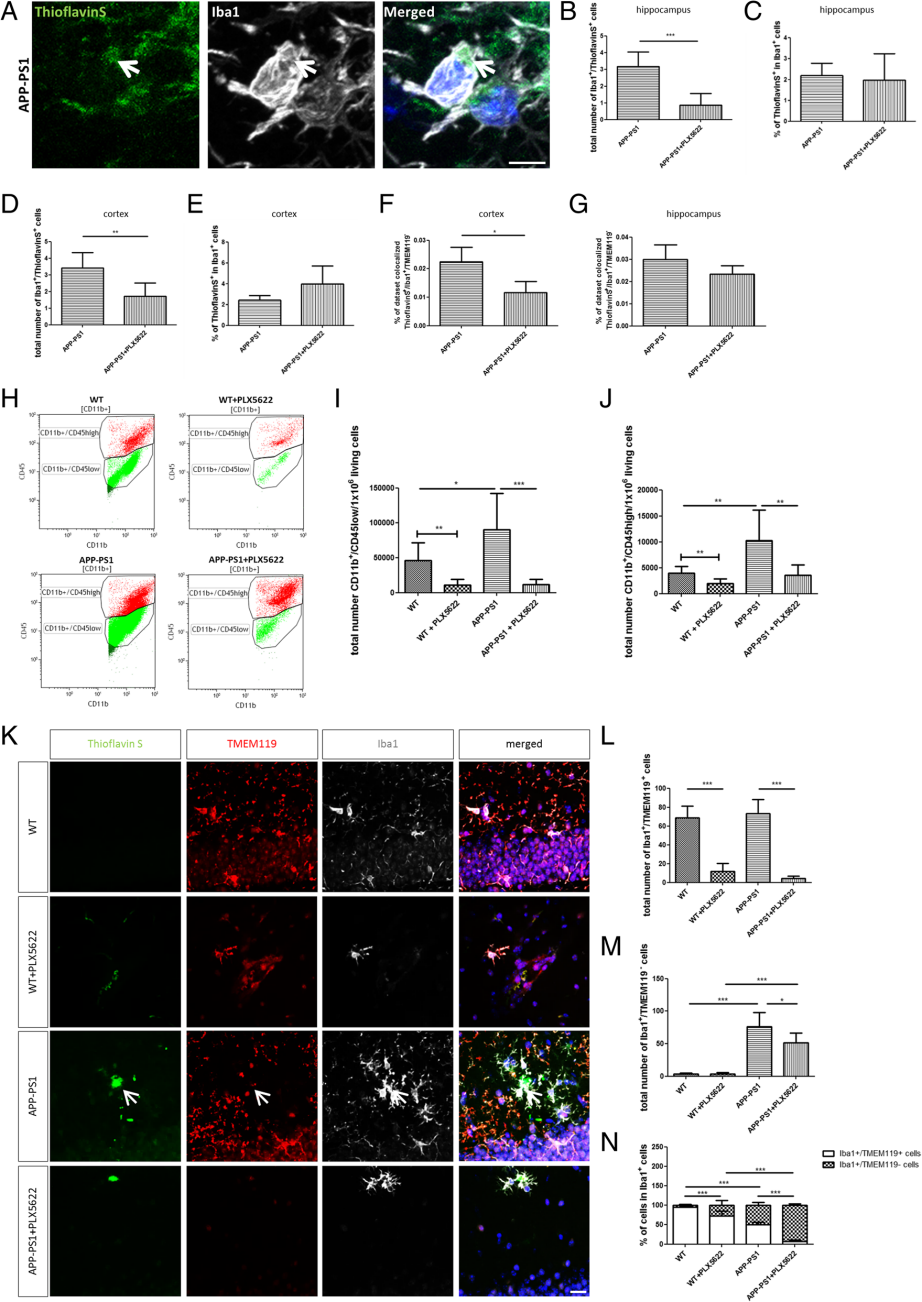
Microglia ablation did not alter amyloid-beta phagocytosis and revealed high numbers of macrophages located at sites of plaques resistant to CSF1R inhibition. **a** Representative image of Iba1^+^ cell at site of plaque with incorporated ThioflavinS^+^ particle in the hippocampus of APP-PS1 mice. The total number of Iba1^+^/ThioflavinS^+^ cells was significantly reduced upon PLX5622 treatment in the hippocampus (**b**) and the cortex (**d**). However, the percentage (%) of Iba1^+^/ThioflavinS^+^ cells from total Iba1^+^ cell counts remained the same in both brain regions of APP-PS1 animals treated with PLX5622 (**c**, **e**). Colocalization analysis with ThioflavinS was performed to analyze the plaque uptake by ThioflavinS^+^/Iba1^+^/TMEM119^−^ cells. There was no observed increase in the amount of engulfed plaque material, represented by the percentage of dataset colocalized with ThioflavinS; however, in the cortex, the percentage of dataset colocalized was significantly reduced in microglia-ablated APP-PS1 mice (**f**). This was not seen in the hippocampus (**g**). Mechanically isolated CD11b^+^ cells were further characterized by their CD45 expression via flow cytometric analysis (**h**) to distinguish microglia (CD11b^+^/CD45^low^, green) from macrophage populations (CD11b^+^/CD45^high^, red). Besides strongly reduced CD11b^+^/CD45^low^ microglia numbers in WT and APP-PS1 animals treated with PLX5622, APP-PS1 mice revealed significantly increased numbers of CD11b^+^/CD45^low^ microglia compared to WT animals (**i**). Analysis of CD11b^+^/CD45^high^ macrophages revealed significantly increased numbers of CD11b^+^/CD45^high^ cells in APP-PS1 compared to WT and treatment with PLX5622 reduced this cell population in WT and APP-PS1 mice (**j**). Using the newly identified microglia marker TMEM119 for detailed immunohistochemical analysis in the hippocampus revealed strong co-localization of Iba1^+^ (white) cells with TMEM119 (red) in WT and WT animals treated with PLX5622; however, Iba1^+^ cells at sites of plaques (green) in APP-PS1 animals and APP-PS1 treated with PLX5622 did not express TMEM119 (**k**, arrow). Quantitative analysis of Iba1^+^/TMEM119^+^ revealed a significant reduction upon PLX5622 treatment in WT and APP-PS1 mice (**l**). Surprisingly, APP-PS1 animals had increased numbers of Iba1^+^/TMEM119^−^ cells that were more resistant to PLX5622 treatment than in WT animals (**m**). (**n**) Calculation of the percentage of Iba1^+^/TMEM119^+^ and Iba1^+^/TMEM119^−^ cells from the total Iba1^+^ cell population. ThioflavinS was used to stain amyloid plaques (green), and Dapi (blue) was used as nucleus stain. Unpaired Student’s *t* test (**b**, **c**, **d** *n* = 6/group; **f**, **g** *n* = 3/group; **j** comparing WT with WT + PLX5622 *n* = 8–9/group;) with Welch’s correction (**e** n = 6/group; **i** comparing WT with WT + PLX5622 *n* = 8–9/group) and one-way ANOVA with Tukey’s multiple comparison test (**i**, **j** *n* = 8–9/group; **l**, **m**, **n** *n* = 6/group) was performed. Scale: 5 μm (**a**), 20 μm (**k**)

Triggered by the lack of PLX5622-mediated alterations in plaque pathology and the lack of effects on learning and memory, we focused on the identity and activity of the PLX5622 treatment-resistant Iba1^+^ and CD11b^+^ cells in the APP-PS1 mice. As Iba1 and CD11b are expressed by CNS resident microglia and by infiltrating macrophages, we first aimed to distinguish between these two cell populations by flow cytometry and by immunohistochemistry. Total mouse brain hemispheres were homogenized, and microglia/macrophage cells were isolated and quantified by flow cytometric analysis using antibodies against CD11b and CD45, where the microglia population is defined by being CD11b^+^/CD45^low^ and the macrophage population by being CD11b^+^/CD45^high^ [45–48]. In APP-PS1 mouse brains, the number of microglia (CD11b^+^/CD45^low^, green) was slightly but significantly higher compared to WT animals (Fig. 4h, i). This was specifically observed in female APP-PS1 mice compared to female WT mice (Additional file 4: Figure S4D). PLX5622 treatment drastically reduced this cell population in both genotypes (Fig. 4h, i). Similarly, the macrophage population (CD11b^+^/CD45^high^) was significantly larger in the brains of APP-PS1 mice compared to WT animals (Fig. 4j) and higher in female APP-PS1 mice compared to female WT mice (Additional file 4: Figure S4G). PLX5622 treatment diminished this cell population, however, with less efficacy compared to the CD11b^+^/CD45^low^ microglia population (Fig. 4j). Also, female and male APP-PS1 mice had higher numbers of CD11b^+^/CD45^high^ cells compared to female and male WT mice; PLX5622 treatment did not decrease this cell numbers in male animals of both genotypes (Additional file 4: Figure S4H). Additionally, female APP-PS1 compared to male APP-PS1 mice had higher numbers of CD11b^+^/CD45^low^ and CD11b^+^/CD45^high^ cell populations (Additional file 4: Figure S4F, I).

To analyze the microglia and macrophage populations in more detail, we performed immunohistochemical analysis using the recently identified microglia-specific marker TMEM119, which in combination with Iba1 immunohistochemistry nicely distinguishes microglia (Iba1^+^/TMEM119^+^) from macrophages (Iba1^+^/TMEM119^−^) in brain sections [48]. The immunohistochemical characterization and quantitative analysis of Iba1^+^/TMEM119^+^ and Iba1^+^/TMEM119^−^ cells in the hippocampus (Fig. 4k) and in the cortex (Additional file 5: Figure S5) revealed that WT and APP-PS1 animals had similar amounts of Iba1^+^/TMEM119^+^ microglia cells and that this population was largely reduced by the PLX5622 treatment (Fig. 4l). In contrast to WT animals, which had only few Iba1^+^/TMEM119^−^ macrophages, APP-PS1 animals contained a higher number of these cells in the hippocampus (Fig. 4m) and cortex (Additional file 5: Figure S5C). Iba1^+^/TMEM119^−^ macrophages colonized mainly areas at sites of ThioflavinS-positive plaques (Fig. 4k, arrow). PLX5622 treatment only slightly diminished this population leaving a high number of Iba1^+^/TMEM119^−^ macrophages in these brain regions (Fig. 4m). Analyzing the percentage of TMEM119^+^ and TMEM119^−^ cell populations in the total Iba1^+^ cell count revealed a small population of Iba1^+^ macrophages in PLX5622-treated WT animals. In contrast, in APP-PS1 animals, approximately 50% of the Iba1^+^ population were macrophages, and most dramatically, in PLX5622-treated APP-PS1 mice, the majority of Iba1^+^ cells was negative for TMEM119 and therefore presumably macrophages (Fig. 4n). This pool of Iba1^+^/TMEM119^−^ macrophages was predominantly observed at sites of amyloid-plaques in APP-PS1 brains and microglia-ablated APP-PS1 brains. Very similar findings were observed in the cortex (Additional file 5: Figure S5). In summary, PLX5622 treatment was highly efficacious to ablate microglia but identified a CSF1R blockage-resistant and plaque-associated Iba1^+^/TMEM119^−^ macrophage population in APP-PS1 animals.

### Iba1^+^/TMEM119^−^ cells expressed the phagocytosis marker CD68 representing a macrophage population presumably involved in amyloid-beta plaque clearance

To determine whether the resistant Iba1^+^ cells take on new and different roles in plaque phagocytosis depending on the presence or absence of microglia, quantitative co-localization analysis was performed to estimate the engulfed amount of plaque material. We counted the number of Iba1^+^ cells that had incorporated ThioflavinS-positive particles (Iba1^+^/ThioflavinS^+^), as an indicator for phagocytosis, in the hippocampus and cortex of APP-PS1 mice and APP-PS1 mice treated with PLX5622 (Fig. 4a, arrow). PLX5622 treatment did significantly reduce Iba1^+^/ThioflavinS^+^ cell numbers in both brain regions (Fig. 4b, d). However, the percentage of Iba1^+^/ThioflavinS^+^ cells in the total Iba1^+^ cell count was the same in PLX5622 and control treated in APP-PS1 mice, indicating that the principal capacity of the Iba1^+^ cells to phagocytose was unaffected by the treatment (Fig. 4c, e). Taken together, this suggests that the PLX5622-resistant Iba1^+^ cell population might be able to compensate the PLX5622 mediated deficiency of phagocytosing microglia in the APP-PS1 mouse brains.

We next analyzed the PLX5622-resistant Iba1^+^ cell population in more detail and estimated the amount of engulfed plaque material, discriminating between macrophages and microglia. The percentage of ThioflavinS colocalized with Iba1, i.e. microglia and macrophages, and colocalized with TMEM119 (microglia) was calculated using Imaris software. There was no general increase in plaque uptake in PLX5622-resistant cells; moreover, in the hippocampus of microglia-ablated APP-PS1 brains, the percentage of colocalization was very similar compared to not-ablated APP-PS1 brains (Fig. 4g). However, PLX5622-resistant Iba1^+^ cells in the cortex (Fig. 4f) showed a significant decrease in colocalization with ThioflavinS upon microglia depletion. Given the fact that there were no alterations in plaque pathology after microglia ablation, we conclude that PLX5622-resistant cells contribute to plaque phagocytosis. However, phagocytotic activity was not elevated in these cells upon microglia ablation in the brains of APP-PS1 mice.

**Fig. 5:**
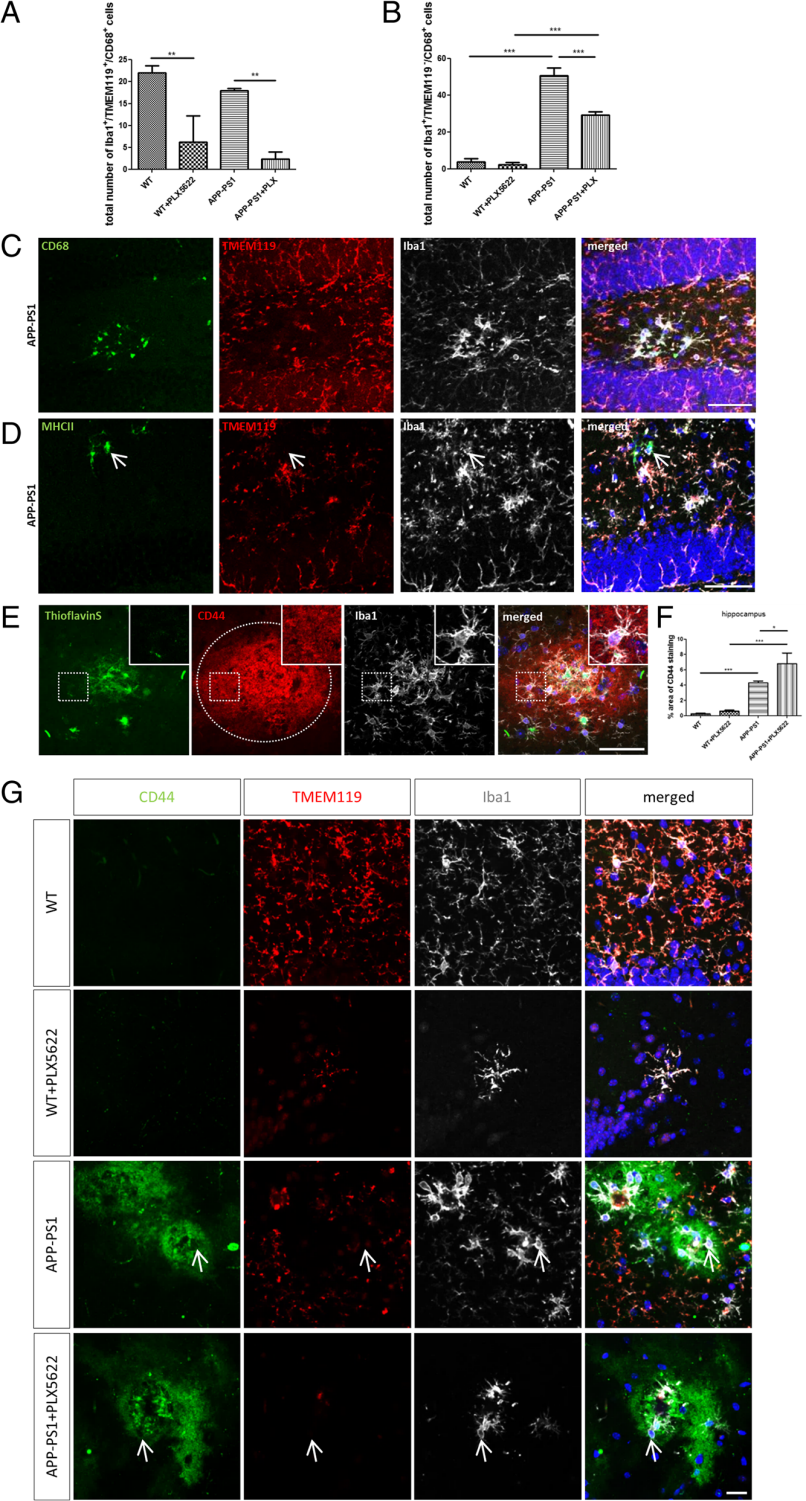
Iba1^+^/TMEM119^−^ cells represent a CD68^+^ macrophage population with peripheral origin highly involved in amyloid-beta phagocytosis. Analysis of CD68 expression in the hippocampus revealed significantly reduced numbers of Iba1^+^/TMEM119^+^/CD68^+^ cells in APP-PS1 and WT animals upon PLX5622 treatment (**a**). Surprisingly, higher numbers of Iba1^+^/TMEM119^−^/CD68^+^ cells were found in APP-PS1 animals compared to WT, although these numbers were slightly reduced in APP-PS1 animals by PLX5622 treatment (**b**). Representative image of CD68 expression in Iba1^+^/TMEM119^−^ cells located at sites of plaque in APP-PS1 mice (**c**). Strong MHCII expression was seen sporadically in Iba1^+^/TMEM119^−^ cells in APP-PS1 mice (**d**, arrow). Accumulation of CD44 staining (red) was observed extracellularly around amyloid depositions (green) as indicated by the doted ellipse, and CD44 staining was seen on Iba1^+^ cells at sites of plaques (**e**, insert). Quantification of percentage (%) area of CD44 staining in hippocampal brain regions revealed barely any staining in WT and WT + PLX5622-treated animals; however, in APP-PS1 and APP-PS1 PLX5622-treated mice, significantly higher expression of CD44 was observed compared to WT controls (**f**). Detailed immunohistochemical analysis revealed increased staining for CD44 at sites of amyloid deposition in areas colonized with Iba1^+^/TMEM119^−^ cells in APP-PS1 and APP-PS1 PLX5622-treated mice (**g**, arrow). ThioflavinS was used to stain amyloid plaques, and Dapi (blue) was used as nucleus stain. One-way ANOVA with Tukey’s multiple comparison test (**a**, **b**, **f** *n* = 3/group) was performed. Scale: 50 μm (**c**, **d**, **e**), 20 μm (**g**)

We further investigated the activity/inflammatory state of microglia (Iba1^+^/TMEM119^+^) and macrophages (Iba1^+^/TMEM119^−^) in the hippocampus (Fig. 5) and cortex (Additional file 6: Figure S6) of WT and APP-PS1 mice by staining for CD68, a classical macrophage and microglia activation marker that is involved in cellular phagocytosis. WT and APP-PS1 mice had the same numbers of CD68^+^ microglia that were equally affected and reduced by the PLX5622 treatment (Fig. 5a). APP-PS1 mice had significantly more CD68^+^ macrophages in the brain compared to WT animals, the latter showing barely any CD68^+^ macrophages (Fig. 5b). PLX5622 treatment reduced this population in APP-PS1 mice, but higher numbers of CD68^+^ macrophages remained in PLX5622-treated APP-PS1 compared to treated WT mice (Fig. 5b). High expression of CD68 was detected in macrophages at sites of amyloid plaques in APP-PS1 mice (Fig. 5c). Besides CD68, the amyloid plaque-associated macrophage population in the APP-PS1 mice sporadically co-localized with MHCII (Fig. 5d) further supporting the macrophage identity of these cells and indicating that these cells are probably involved in antigen presentation. Similar findings were observed in the cortex (Additional file 6: Figure S6).

To further substantiate the peripheral origin of Iba1^+^/TMEM119^−^ macrophage cells characterized in this work, we used CD44 as recently described marker to distinguish between resident and infiltrating immune cells in the brain [46]. Surprisingly, we found very prominent staining for CD44 in APP-PS1 and APP-PS1 PLX5622-treated mice around amyloid-beta plaques in the hippocampus (Fig. 5e) and the cortex (Additional file 6: Figure S6E). CD44 was localized extracellularly around the amyloid plaques and at Iba1^+^ cells closely attached to amyloid plaques (Fig. 5e, insert). Quantification of CD44 staining revealed barely any CD44 staining in WT or WT PLX5622-treated mice; however, CD44 staining was significantly higher in the hippocampus (Fig. 5f) and cortex (Additional file 6: Figure S6F) of APP-PS1 and APP-PS1 PLX5622-treated mice. Triple staining with Iba1 and TMEM119 revealed intense staining for CD44 at sites of plaques, where Iba1^+^/TMEM119^−^ cells were located (Fig. 5g, arrow). Very similar findings were observed in cortical brain regions (Additional file 6: Figure S6). These data further suggest that Iba1^+^/TMEM119^−^ cells have peripheral origin and that the extracellular adhesion molecule CD44 might play a pivotal role in leukocyte homing from the periphery to the CNS and to the sites of inflammation in AD.

### Elevated levels of CD8^+^ T-cells in brains of APP-PS1 mice and increased CD8^+^ T-cell homing to microglia-ablated APP-PS1 mouse brains

**Fig. 6:**
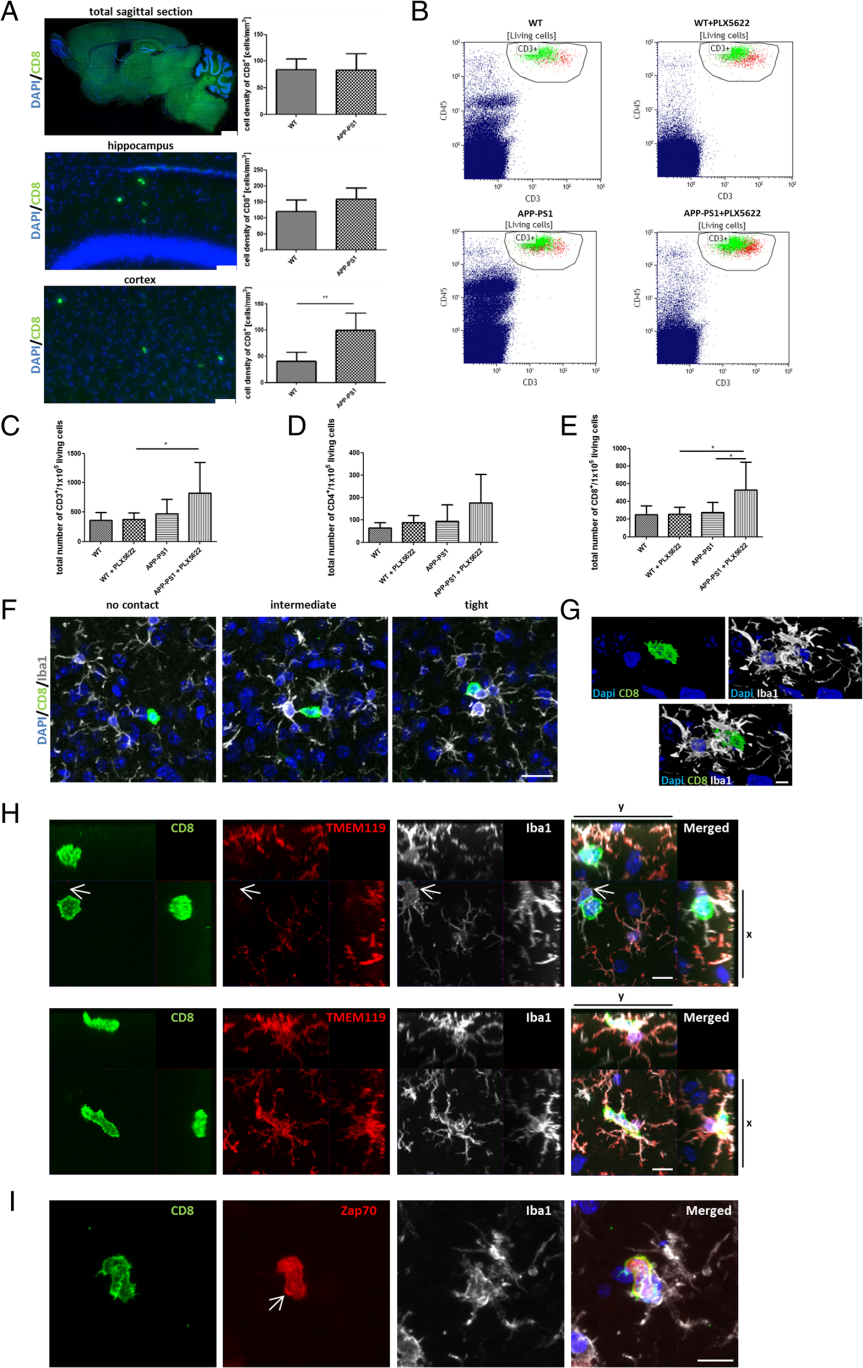
Microglia ablation in APP-PS1 mice resulted in increased CD3^+^/CD8^+^ T-cell numbers in the brain, and CD8^+^ T-cell numbers were highly increased in cortical brain regions of APP-PS1 mice interacting with Iba1^+^/TMEM119^+^ and Iba1^+^/TMEM119^−^ cells. Detailed immunohistochemical analysis of CD8^+^ T-cell numbers (green) in brains of APP-PS1 mice revealed similar CD8^+^ T-cell numbers compared to WT when analyzing total sagittal brain sections; however, CD8^+^ T-cell numbers were significantly increased in cortical brain regions of APP-PS1 mice compared to WT (**a**). T-cells were mechanically isolated from total brain hemispheres and quantitatively analyzed by flow cytometry after ablation of microglia cells (**b**, gated for single-living CD45^+^/CD3^+^ cells; CD4^+^ red, CD8^+^ green). The total number of CD45^+^/CD3^+^ T-cells was significantly increased in APP-PS1 mice treated with PLX5622 compared to WT-treated mice (**c**). CD3^+^/CD4^+^ T-cell numbers trend to be increased in APP-PS1 mice treated with PLX5622 (**d**). CD3^+^/CD8^+^ T-cell numbers were significantly increased in APP-PS1 mice treated with PLX5622 compared to APP-PS1 and WT PLX5622-treated mice (**e**). CD8^+^ T-cells in APP-PS1 mice were located directly in the brain parenchyma and observed in different interaction types with Iba1^+^ cells (white), showing either no contact, intermediate, or very tight interaction with brains Iba1^+^ cells (**f**). Performing confocal microscopy, we observed very tight cell to cell interactions with Iba1^+^ cells (**g**) and orthogonal projections showed CD8^+^ T-cells associated with Iba1^+^/TMEM119^+^ and as well with Iba1^+^/TMEM119^−^ cells in APP-PS1 mice (arrow, **h**). CD8^+^ T-cells that tightly associated to Iba1^+^ cells showed high expression of Zap70 (red) at sites of the cell membrane, indicating immune synapse formation, and activation of the T-cell receptor complex (arrow, **i**). Dapi (blue) was used as nucleus stain. One-way ANOVA with Tukey’s multiple comparison test (**c**, **d**, **e** *n* = 8–9/group) and unpaired Student’s *t* test (**a**, *n* = 5/group) was performed. Scale: 1 mm and 50 μm (**a**), 20 μm (**f**), 10 μm (**h**, **i**), 5 μm (**g**)

Apparently, lymphocytes are one of the biggest peripheral immune cell populations observed in the CNS of healthy adult mice [46]. Besides, T-cells are reported to enter the brain parenchyma of AD-transgenic mice [31]. We recently identified a specific T-cell population highly associated and presumably interacting with resident microglia in the brains of APP-PS1-transgenic mice [37]. Here, we first quantified the number of CD8^+^ T-cells present in the brains of WT and of APP-PS1 mice via histology in total sagittal brain sections. While the numbers did not differ for the total brain, there was a significantly higher number of CD8^+^ T-cells in the cortex of APP-PS1 mice compared to WT controls (Fig. 6a). To address now the question, if T-cell infiltration is microglia dependent, we made further use of the present PLX5622-mediated microglia depletion experiment and analyzed the numbers and identity of T-cells in the brain of WT and APP-PS1 mice where microglia cells were ablated. We first isolated T-cells from total brain hemispheres of WT and APP-PS1 mice revealing no difference in the number of CD3^+^, CD3^+^/CD4^+^, or CD3^+^/CD8^+^ T-cells in APP-PS1 mice compared to WT (Fig. 6b–e) confirming the histology data from above. A gender-specific analysis of the flow cytometric data revealed increased CD3^+^, CD3^+^/CD4^+^, and CD3^+^/CD8^+^ T-cell numbers in female compared to male APP-PS1 mice (Additional file 7: Figure S7C, F, I), and increased cell numbers were found in female APP-PS1 mice when specifically compared to female WT mice (Additional file 7: Figure S7A, D, G). Surprisingly, ablation of microglia caused a significant increase in the numbers of CD3^+^, more specifically CD3^+^/CD8^+^ T-cells, exclusively in brains of APP-PS1 and not WT mice treated with PLX5622 (Fig. 6b, c, e). CD4^+^ T-cells were slightly but not significantly increased in PLX5622-treated APP-PS1 mice. Microglia ablation apparently allowed specifically CD3^+^/CD8^+^ T-cell homing to the APP-PS1 brain. Vice versa, this let suggest that microglia in APP-PS1 mice might somehow inhibit specifically CD3^+^/CD8^+^ T-cell recruitment to the brain. To test if the herein observed increase in CD8^+^ T-cell numbers might arise from proliferation of brain resident T-cells, we additionally stained for expression of the proliferation cell nuclear antigen (PCNA). CD8^+^ T-cells in the brain of PLX5622-treated or untreated APP-PS1 animals did not show expression of PCNA indicating no clonal expansion and no local generation of these cells in the brain (Additional file 8: Figure S8). To investigate the CD8^+^ T-cells in the context of microglia in more detail, we analyzed their spatial relation to Iba1^+^ cells in APP-PS1 brains. CD8^+^ T-cells were in different proximities and interactions with Iba1^+^ cells. They either had barely any cell to cell contact, some interaction at the level of the cellular processes, or a very close and tight interaction with Iba1^+^ cells (Fig. 6f). In the latter, high magnification of confocal z-stacks and 3D reconstruction revealed very close cell to cell communication of CD8^+^ T-cells with Iba1^+^ cells (Fig. 6g). To address the question if CD8^+^ T-cells are interacting only with microglia or as well with the herein described macrophage population in APP-PS1 mice, we performed triple staining with Iba1 and TMEM119 and could demonstrate that CD8^+^ T-cells form tight interaction with both cell types (Fig. 6h).

To further confirm this immune cell interaction present in APP-PS1 brains, we stained for Zap70, an essential kinase for T-cell receptor function and immune synapse formation. CD8^+^ T-cells in the brain were mostly positive for Zap70, and Zap70 staining was very prominent on the cell membrane and clustered at sites of interaction with Iba1^+^ cells (Fig. 6i, arrow) suggesting a functional interaction between the two cell types. Besides CD8^+^ T-cells, we qualitatively analyzed the brains of APP-PS1 mice for CD4^+^ T-cells via histology. As expected from the flow cytometric data, CD4^+^ T-cells were observed less frequently than CD8^+^ T-cells, even though they did express the immune synapse marker Zap70 (Additional file 9: Figure S9). In summary, microglia ablation resulted in increased CD3^+^/CD8^+^ T-cell homing to sites of inflammation specifically in the brain of APP-PS1 mice, suggesting that the present microglia in APP-PS1 mice might block adaptive immune responses along AD pathology.

### Microglia depletion has profound consequences on gene transcription of pro- and anti-inflammatory and phagocytosis-relevant genes in the brain

**Fig. 7:**
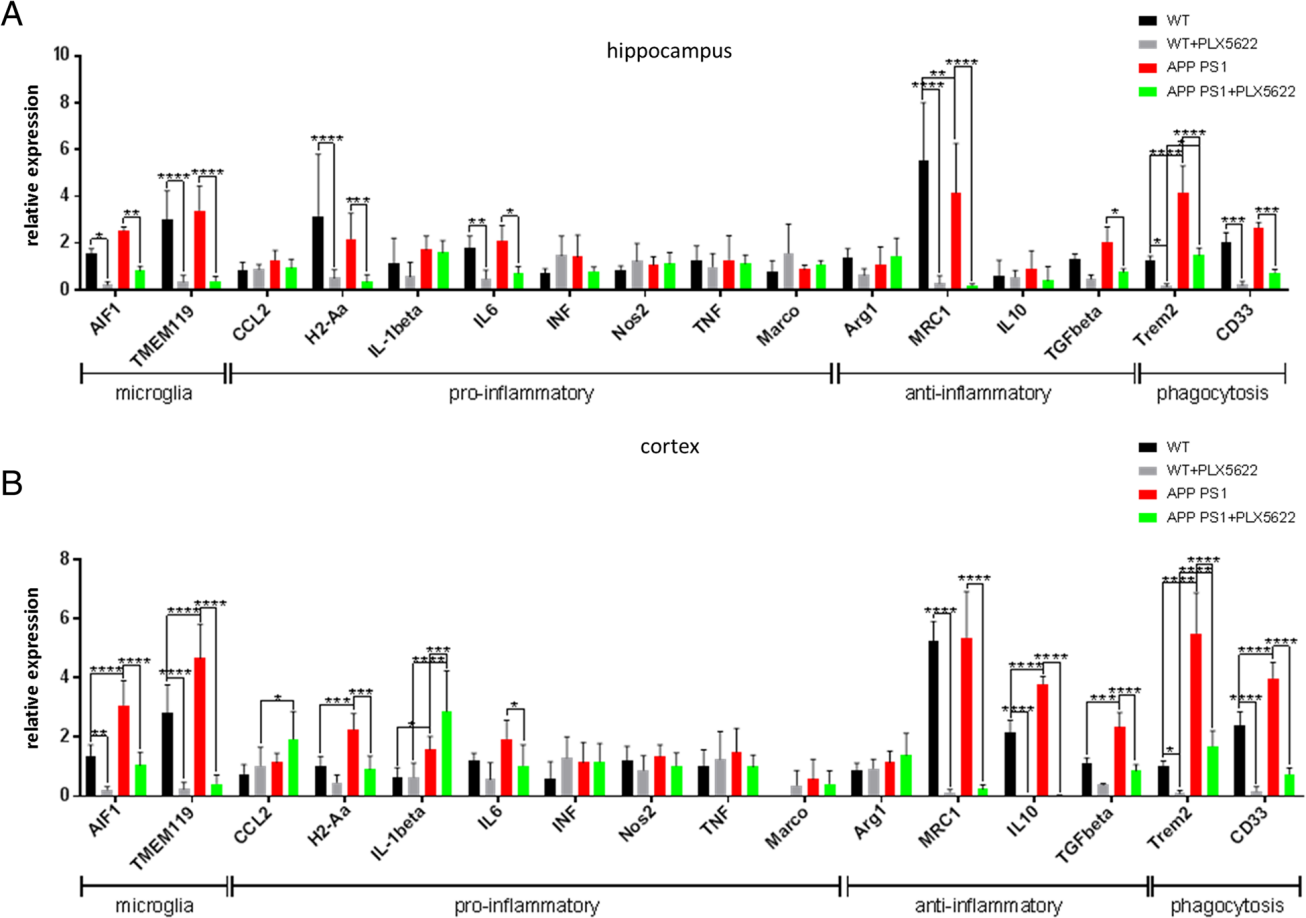
Gene expression analysis in the brain after PLX5622 treatment. Gene transcription was successfully downregulated for several microglia, pro-inflammatory, anti-inflammatory, and phagocytosis-relevant genes upon microglia ablation in the hippocampus (**a**) and cortex (**b**) of WT and APP-PS1 mice. Two-way ANOVA with Tukey’s multiple comparison test (*n* = 6–8/group) was performed

To analyze the herein described rather complex cellular effects of PLX5622 treatment on the brain’s immunological micro-environment, we performed mRNA expression analysis of genes specific for microglia, for a pro- or anti-inflammatory micro-milieu, and for phagocytosis (Fig. 7a, b). In APP-PS1 compared to WT mice, expression of the microglia genes AIF1 (=Iba1) and TMEM119 was only slightly higher in the hippocampus (Fig. 7a) but significantly (about two-fold) higher in the cortex (Fig. 7b) correlating with the histological and flow cytometric findings (Fig. 2). Along this line, APP-PS1 brains had higher expression levels of the phagocytosis-relevant genes Trem2 and CD33. APP-PS1 brains showed a more pronounced elevation of some pro-inflammatory (H2-Aa = MHCII, IL-1beta, CCL2), as well as some anti-inflammatory (IL10, TGF-beta) genes in the cortex compared to the hippocampus. The 28-day PLX5622 treatment resulted in a highly diminished but not completely erased expression of AIF1 and TMEM119 in the hippocampus and cortex in both genotypes, which again correlates with the histological and flow cytometric findings. The pro-inflammatory genes H2-Aa and IL6 were significantly lower in the hippocampus of APP-PS1 and WT animals after treatment with PLX5622. This effect was less pronounced in the cortex. Moreover and surprisingly, gene expression levels of CCL2 and of IL-1beta were higher in the PLX5622-treated APP-PS1 cortex compared to control-treated and compared to WT cortex. The elevated expression of CCL2 is especially of interest, as it is known to be a major chemoattractant for monocytes and might explain the prominent presence of monocytes after PLX5622 treatment in the APP-PS1 brains. Microglia ablation affected also the gene expression of the anti-inflammatory molecules MRC1 (=CD206) and TGFbeta. In the hippocampus, MRC1 and TGFbeta gene expression was massively reduced upon PLX5622 treatment in WT and APP-PS1 animals. This effect was even more pronounced in the cortex, where also IL10 expression was almost completely erased by the PLX5622 treatment. PLX5622 treatment led to an enormous reduction in Trem2 expression, however more effective in WT than in APP-PS1 mice. CD33 was also massively lower in PLX5622-treated WT and APP-PS1 mice compared to the control animals. In summary, APP-PS1 mice had a higher expression of microglia- and phagocytosis-specific genes compared to WT animals, as well as an alteration in the pattern of pro-inflammatory as well as anti-inflammatory proteins. PLX5622 treatment resulted in diminished expression of the microglia- and phagocytosis-specific genes in WT as well as APP-PS1 brains and, except for a higher expression of CCL2 and of IL-1beta in the cortex, resulted in a reduction of the expression of some pro-inflammatory but mainly and more pronounced in the reduction of anti-inflammatory cytokine expression.

## Discussion

In the present work, we demonstrate that a 28-day treatment with PLX5622 resulted in massive reduction of microglia cell numbers and a decrease of microglia-related gene expression in the brains of WT and APP-PS1 mice. Furthermore, we show that APP-PS1 mice harbor a specific Iba1^+^/TMEM119^−^ and CD68^+^ macrophage population resistant to CSF1R treatment presumably involved in plaque clearance. Ablation of microglia revealed the existence of these cells at sites of amyloid plaques, and colonization by macrophages appeared with a strong CD44 staining at sites of amyloid-plaques, which together with the elevated CCL2 expression points towards a milieu that is highly attractive for peripheral immune cells. Indeed, microglia depletion in APP-PS1 animals increased the number of CD3^+^/CD8^+^ T-cells specifically in the brains of APP-PS1 mice. These T-cells expressed the immune synapse marker Zap70 and additionally were in close association with remaining microglia and macrophages. Therefore, we assume that in AD pathology, microglia restrain the brain from an adaptive immune response and act in that sense primarily anti-inflammatory. In their absence, macrophages might function as important linchpin and connect innate to adaptive immune responses by recruiting T-cells to the AD brain. Overall, microglia depletion led to a diminished expression of anti-inflammatory cytokines in the APP-PS1 mice shifting the balance towards a pro-inflammatory milieu.

Over the past years, there is emerging evidence that the brain is not immunologically isolated from the peripheral immune system (reviewed in [59, 60]). The brain and its specific compartments, i.e., the meninges and choroid plexus, but also the brain parenchyma itself is highly immune-competent and harbors a diversity of peripheral-derived immune cells comparable to the blood [46]. Bone marrow-derived monocytes and macrophages were observed to infiltrate the brains of transgenic AD mice [27] and are controversially discussed to participate in amyloid clearance (reviewed in [28–30] and [61]). Even though infiltration of macrophages in brains of transgenic AD mice was already observed by others ([27], reviewed in [23]), the clear presence of Iba1^+^ cells lacking the microglia-specific marker TMEM119 (Iba1^+^/TMEM119^−^) located at sites of amyloid plaques in transgenic AD mouse brains is to our knowledge not yet reported. Here, we show for the first time a specific TMEM119^−^ macrophage population colonizing at sites of the amyloid plaques in APP-PS1 mice. The recently identified specific microglia marker TMEM119 developed by Bennet et al. [48] allowed us to clearly distinguish the microglia population from the macrophage population in the transgenic AD brains. TMEM119 reliably stains microglia in the human brain, while macrophages (Iba1^+^/CD68^+^) at sites of necrotic lesion in human multiple sclerosis (MS) brains do not express TMEM119 [62]. How microglia cells can be distinguished from peripheral-derived macrophage populations in the brain and how these two cell types differ from their genetic profile and marker expression is currently discussed [63, 64]. For example, only microglia and not macrophages fully regain their cellular identity, when transplanted in the brain of microglia-deficient mice. Bennett et al. demonstrated that brain environment and microglia ontogeny are crucial for keeping cellular identity and characteristics. However, macrophages of different peripheral origin transplanted to the microglia-depleted brain are able to engraft and become a microglia-like cell population, still keeping specific ontogeny markers, but hard to distinguish from brain resident microglia cells [63]. To what extend macrophages that once entered the brain can turn into microglia cells or keep their cellular identity and if this has any functional consequences especially under neurodegenerative conditions is still scarce [64]. Nevertheless, the herein presented data are in line with the current literature and show the spatial presence of this non-microglia population in the brain at sites of inflammation in the APP-PS1 transgenic mice.

There are various reports on microglia depletion targeting the CSF1R receptor in mice [38, 39, 55, 56, 65, 66]. In these studies, CSF1R signaling is either blocked using small molecular inhibitors [55, 56], CSF1R^−^/^−^ knockout mice [67, 68], or genetically modified inducible microglial reporter mice, where transient microglia ablation is achieved by diphtheria toxin injections [65] or by ganciclovir administration in suicide gene herpes simplex virus thymidine kinase (HSVTK)-carrying mice (reviewed in [69] and [70]). Microglia ablation specifically in AD mice resulted in several beneficial effects on AD pathology such as a reduction in dendritic spine and neuronal loss as well as behavioral alterations, e.g. improvement in contextual memory [38, 39]. However, although neuroinflammation was highly dampened in these microglia-ablated mice, amyloid plaque pathology was not altered by CSF1R inhibition [38, 39]. Prokop et al., who conditionally ablated microglia cells with ganciclovir treatment using a CD11b-HSVTK transgenic AD mouse model, analyzed the capability of peripheral myeloid cells in repopulating the brain and their potential in amyloid-beta plaque phagocytosis. They demonstrated that after microglia depletion, the brains were nearly completely exchanged by peripheral myeloid cells and the overall amyloid-beta burden was unchanged in repopulated brains [61]. These data are in line with our data, indicating that peripheral-derived macrophages might compensate functions of microglia.

Besides monocytes/macrophages, lymphocytes resemble a big proportion of peripheral immune cells [46, 71]. Specifically, T-cells are reported to infiltrate the CNS in higher numbers during normal brain aging ([71], reviewed in [72]) and under pathological conditions as for example after viral infections (reviewed in [73]). Also, under neurodegenerative conditions such as Parkinsons’s disease (PD) [74], amyotrophic lateral sclerosis (ALS) (reviewed in [75]), stroke (reviewed in [76]), and autoimmune diseases such as MS and its animal model experimental autoimmune encephalomyelitis (reviewed in [77]), increased numbers of lymphocytes were observed in the brain. Contribution of lymphocytes to AD is less evident; however, there are few reports on mostly CD3^+^/CD4^+^ T-cells or regulatory T-cells (Tregs) infiltrating the brains of transgenic AD mice with already advanced pathology [31, 36]. The presence of T-cells was first mentioned in human AD brains in 1988 [32, 33]. In terms of their functional relevance, increased CD3^+^ T-cell numbers were recently reported to correlate with Tau pathology rather than amyloidosis in humans [34]. The exact functional role of T-cells in the context of AD is not known at all, and ablation of the complete T-cell population (CD3^+^ cells) [78], of regulatory T-cells (Treg) [25, 36]), or immuno-deficiency in AD mice showed rather conflicting results [24]. Therefore, it is still under debate, if T-cells are beneficial or detrimental in AD pathology.

In the present work, we could demonstrate that CD8^+^ T-cells form tight cell to cell contact with microglia and macrophages in the brain of transgenic AD mice and that this interaction is highlighted by the formation of an immune synapse as indicated by translocation of Zap70 to the T-cell surface [79]. Zap70 is an essential kinase for T-cell receptor (TCR) signaling and important for the formation of immune synapses upon interaction of T-cells with antigen presenting cells (APC) [80, 81]. Indeed, this is the first presentation of a direct microglia (as an APC) -T-cell interaction in vivo in the context of AD. Our data strengthens the hypothesis that the adaptive immune system is highly involved in AD pathology and crosstalks with innate immune processes in the brain. In our study, we primarily focused on CD8^+^ T-cell populations. Normally, CD8^+^ T-cells have cytotoxic effector function eliminating infected target cells or tumor cells (reviewed in [82]); their role in neurodegenerative diseases is unclear but might open new research avenues and treatment options for AD. We demonstrate that after microglia depletion, high numbers of CD3^+^/CD8^+^ T-cells were recruited to the brain, specifically in the APP-PS1 animals, assuming that under healthy conditions, T-cell recruitment is kept at a certain baseline level. Since WT mice do not harbor high amounts of macrophages in the brain, the herein described remaining Iba1^+^/TMEM119^−^ macrophages at sites of plaques in the APP-PS1 mice might be responsible for the increased T-cell homing.

A key molecule probably responsible for leukocyte homing to the brain is CD44. CD44 is the major surface hyaluronan (HA) receptor originally expressed on lymphocytes and epithelial cells, and its primary function is to mediate interaction of immune cells with the endothelium [83]. CD44 is involved in intercellular adhesion and in cell signaling. In the CNS, CD44 is expressed by glial cells and neurons including their dendritic and axonal processes (reviewed in [84]). In human AD patients, increased CD44 gene expression in lymphocytes was observed, implicating strong participation of CD44 in peripheral immune responses along AD pathology [85]. Furthermore, astrocytes in human AD brains were described to express CD44 [86], and an astrocytoma cell line exposed to amyloid-beta increased CD44 expression in vitro [87]. Additionally, CD44 is highly involved in leukocyte trafficking to inflamed tissue ([88], reviewed in [89]) and is upregulated after activation of T-cells, remaining on the surface of memory T-cells [90]. CD44 is a key molecule that directs T-cells to the sites of inflammation [91]. Most recently, Korin et al. showed by high-dimensional CyTOF mass cytometry that peripheral-derived immune cells in the brain can be distinguished from brain resident immune cells by expression of CD44. Brain resident myeloid cells (CD11b^+^) that were positive for CD44 lacked the expression of TMEM119, and the authors therefore claimed that CD44 can be used in the brain to detect infiltrating immune cells and distinguish them from brain resident cells [46]. In agreement with this data, we observed increased staining for CD44 around amyloid-beta plaques, strongly suggesting that high amounts of extracellular CD44 might be involved in leukocyte attraction to the plaques. To understand the exact function of CD44 and its role in AD pathology, further studies have to be performed.

The treatment with PLX5622 did reduce microglia numbers and lowered the expression of some pro-inflammatory genes but strongly diminished the expression of anti-inflammatory genes in certain brain regions as previously reported by others [39]. Thus, our data suggest that microglia cells might be responsible for creating an inflammatory milieu in the brain in the context of AD. We showed increased expression of genes for pro-inflammatory molecules (H2-Aa = MHCII, IL-1beta) and anti-inflammatory molecules (IL10, TGFbeta) specifically in the cortex of APP-PS1 mice compared to WT animals. Whereas microglia ablation highly reduced the anti-inflammatory gene transcripts or decreased them at least to WT levels, a pro-inflammatory signature by CCL2 and IL-1beta remained in the microglia-ablated cortex of APP-PS1 mice (Fig. 7b). CCL2 is also referred to as monocyte chemoattractant protein 1 (MCP1), a strong chemoattractant for monocytes and memory T-cells (reviewed in [92]) and has been identified as a blood-derived aging factor [93]. In human genome-wide association studies of prodromal AD [94] and AD patients with mild cognitive impairment (MCI), high amounts of CCL2 were detected in the CSF and CCL2 levels correlated with a faster rate of cognitive decline in the analyzed patient cohort [95]. CCL2 can be produced by a variety of cells including endothelial cells, fibroblasts, astrocytes, and microglia but also by monocyte/macrophages (reviewed in [96]). High levels of CCL2 gene transcripts remained specifically in the cortex of APP-PS1 microglia-ablated brains indicating that CCL2 is not exclusively produced by microglia. A possible explanation might be that the remaining macrophage population itself produces high amounts of CCL2 to attract the herein described CD8^+^ T-cell population and to further link the innate with the adaptive immune response in the brain.

Nevertheless, our herein performed gene expression analysis has its limitations. Gene expression was analyzed from total hippocampal and cortical brain regions, and therefore it cannot be excluded that other cell populations, e.g. astrocytes, might compensate microglia-dependent loss of signal molecules. Furthermore, microglia cell death itself might cause changes in gene expression. However, since microglia cells are specifically killed with PLX5622 treatment in WT and APP-PS1 mice, the observed reduction in microglia and inflammatory-specific genes highly suggests microglia cells as the main producer of inflammation in the CNS. Our gene expression analysis illustrated increased gene expression of anti-inflammatory molecules IL-10 and TGFbeta in APP-PS1 cortex samples (Fig. 7b). Chakrabarty et al. showed that an anti-inflammatory treatment with IL10 resulted in decreased amyloid-beta phagocytosis, increased plaque burden, and impaired memory function in transgenic AD mice [19]. In line with this data, Guillot-Sestier et al. showed elevated IL10 signaling pathways in human AD brains and analysis of a transgenic AD mouse model with IL10 knockdown (APP/PS1 + IL10^−/−^) demonstrated mitigation of various AD characteristics and again promotes microglia amyloid-beta phagocytosis in these mice [97]. If this hypothesis holds, a fine-tuned shift from anti-inflammatory microglia towards a more pro-inflammatory phenotype might be a beneficial AD treatment option to be validated and is discussed in the field [98].

## Conclusion

Our data suggest that microglia in the brains of APP-PS1 transgenic mice promote an anti-inflammatory milieu and limit T-cell recruitment to the brain, therefore restricting adaptive immune responses in the brain. Ablation of microglia cells revealed a macrophage population present at sites of amyloid plaques highly involved in amyloid-beta clearance and presumably playing a key element in linking innate with adaptive immune responses along AD pathology. The presence of peripheral myeloid and lymphoid cell populations in the brain and their interaction with microglia is fascinating and strengthens the idea that the development of AD pathology is no longer brain restricted but also driven by the adaptive immune system. Further investigations on the functional relevance of each corresponding immune cell population and their interaction with microglia in the brain are necessary. Understanding the immune cell interactions present in the brain during the cause of AD might allow new treatment options for AD pathology.

## Additional files

Additional file 1: **Figure S1.** Qualitative immunohistochemical staining for CSF1R receptor (red) in mouse brain cortex showed high expression in Iba1^+^ cells (white) in all studied groups. ThioflavinS was used to stain amyloid plaques (green) and Dapi (blue) was used as nucleus stain. Scale: 50 μm. (TIF 3837 kb)

Additional file 2: **Figure S2.** Microglia ablation has no impact on learning behavior and did not improve learning deficits in APP-PS1 animals. Morris Water Maze (MWM) test for spatial learning and memory was performed and the total distance the animals moved to reach the platform was calculated as measure for learning improvement. (A) All animals moved with the same swim speed. There was a significant difference between the total distances traveled to reach the platform from day 2 to day 5 in APP-PS1 mice compared to WT animals (B). In WT mice PLX5622 treatment has no impact on the total distance the animals moved to reach the platform (C). Microglia ablation in APP-PS1 mice did not improve learning deficits compared to untreated APP-PS1 mice (D) or WT PLX5622 treated mice (E). Spatial memory was tested on day 6 after platform removal and the duration of the animals in the original platform quadrant was measured (F). There was no significant differences comparing all 4 groups, however WT PLX5622 treated mice spent decreased time in the original platform quadrant when only compared to WT mice (F). Representative track visualization of the total distances traveled at day 5 in MWM test (G). Data are shown as mean with SEM (A-F). Two-way ANOVA with Bonferroni Post-test was performed (A-E, *n* = 9/group) and One-way ANOVA with Tukey’s Multiple Comparison test or Unpaired Student’s T-test were performed comparing only WT with WT + PLX5622 (F, n = 9/group). (TIF 536 kb)

Additional file 3: **Figure S3.** Behavioral data of the Morris Water Maze test were analyzed for gender specific differences: Microglia ablation had no sex-specific impact on learning behavior in female (A) or male (B) WT mice and there was no gender-specific difference in the total distance WT mice moved to reach the platform (C). APP-PS1 female (D) and male (E) mice traveled higher distances to reach the platform compared to female and male WT mice, however no gender differences were observed in APP-PS1 mice (F). Microglia ablation in APP-PS1 mice did not improve learning deficits in female or male mice compared to either untreated APP-PS1 mice (G, H) or WT PLX5622 treated mice of corresponding gender (J, K). There was no difference in  the distance moved between female and male WT  mice treated with PLX5622 (I). Female and male APP-PS1 microglia ablated mice showed no sex difference in the distance moved to reach the platform (L). Spatial memory was tested on day 6 after platform removal and the duration of the animals in the original platform quadrant was measured. A trend for reduced memory of the spatial platform location was observed in female WT PLX5622 and female APP-PS1 PLX5622 treated mice compared to respective controls (M), but no significant difference was observed in male PLX5622 treated animals (N). Comparison of the duration in the platform quadrant in female versus male mice for the single studied groups showed a significant reduction in spatial memory in male APP-PS1 mice compared to female APP-PS1 (O). Data are shown as mean with SEM (A-O). Two-way ANOVA with Bonferroni Post-test (A-L, *n* = 4–5/group), One-way ANOVA with Tukey’s Multiple Comparison test (M, N, n = 4–5/group) and Unpaired Student’s T-test for comparison of female versus male in the respective groups (O, n = 4–5/group) were performed. (TIF 867 kb)

Additional file 4: **Figure S4.** Detailed analysis for gender-specific differences of flow cytometric data from brain isolated microglia/macrophage populations: PLX5622 treatment reduced CD11b^+^ cell numbers in the brain of female (A) and male (B) animals, however specifically female APP-PS1 mice had higher numbers of CD11b^+^ cells compared to female WT mice. Gender-specific differences were observed in APP-PS1 mice, where the females had higher numbers of CD11b^+^ cells compared to male APP-PS1 mice (C). Similar results were obtained from microglia cell numbers (CD11b^+^/CD45^low^) of APP-PS1 mice with increased numbers of microglia in female APP-PS1 compared to female WT mice and compared to male APP-PS1 animals (D-F). Macrophage numbers (CD11b^+^/CD45^high^) where significantly increased in female APP-PS1 compared to female WT mice and were reduced upon PLX5622 treatment in both genotypes (G). Also male APP-PS1 mice had increased numbers of CD11b^+^/CD45^high^ cells compared to male WT mice, however PLX5622 treatment did not reduce macrophage numbers in male animals of both genotypes (H). Higher numbers of macrophages were already detected in the brains of female APP-PS1 mice compared to male APP-PS1 animals (I). One-way ANOVA with Tukey’s Multiple Comparison test (A, B, D, E, G, H n = 4–5/group) and Unpaired Student’s T-test (A, C, D, F, G, H, I) with Welch’s correction (B, E) for comparison of only two groups were performed (n = 4–5/group). (TIF 727 kb)

Additional file 5: **Figure S5.** Using the newly identified microglia specific marker TMEM119 for detailed immunohistochemical analysis in the cortex revealed strong co-localization of Iba1^+^ (white) cells with TMEM119 (red) in WT and WT animals treated with PLX5622 (A). However, Iba1^+^ cells at sites of plaques (green) in APP-PS1 and PLX5622 treated APP-PS1 animals did not express TMEM119. Quantitative analysis of Iba1^+^/TMEM119^+^ revealed a significant reduction in Iba1^+^/TMEM119^+^ cell numbers upon PLX5622 treatment in WT and APP-PS1 mice (B). Surprisingly, APP-PS1 animals had increased numbers of Iba1^+^/TMEM119^−^ cells that were more resistant to PLX5622 treatment than in WT animals (C). Calculation of the percentage of Iba1^+^/TMEM119^+^ and Iba1^+^/TMEM119^−^ cells from the total Iba1^+^ cell population (D). ThioflavinS was used to stain amyloid plaques (green) and Dapi (blue) was used as nucleus stain. One-way ANOVA with Tukey’s Multiple Comparison Test (B, C, D *n* = 6/group) was performed. Scale: 20 μm (A). (TIF 1310 kb)

Additional file 6: **Figure S6.** Iba1^+^/TMEM119^−^ cells represent a CD68^+^ macrophage population with peripheral origin highly involved in amyloid-beta phagocytosis. Analysis of CD68 expression in the cortex revealed significantly reduced numbers of Iba1^+^/TMEM119^+^/CD68^+^ cells in APP-PS1 and WT animals upon PLX5622 treatment (A). Surprisingly, higher numbers of Iba1^+^/TMEM119^−^/CD68^+^ cells were found in APP-PS1 animals compared to WT, although these numbers were slightly reduced in APP-PS1 animals by PLX5622 treatment (B). Representative image of CD68 expression in Iba1^+^/TMEM119^−^ cells located at sites of plaque in APP-PS1 mice (C). Strong MHCII expression was seen sporadically in Iba1^+^/TMEM119^−^ cells in APP-PS1 mice (D, arrow). Accumulation of CD44 staining (red) was observed extracellularly around amyloid depositions (green) as indicated by the doted ellipse and CD44 staining was seen on Iba1^+^ cells at sites of plaques (E, insert). Quantification of percentage (%) area of CD44 staining in hippocampal brain regions revealed barely any staining in WT and WT + PLX5622 treated animals, however in APP-PS1 and APP-PS1 + PLX5622 treated mice significantly higher expression of CD44 was observed compared to WT or WT + PLX5622 animals (F). Detailed immunohistochemical analysis revealed increased staining for CD44 at sites of amyloid deposition in areas colonized with Iba1^+^/TMEM119^−^ cells in APP-PS1 and APP-PS1 PLX5622 treated mice (G, arrow). ThioflavinS was used to stain amyloid plaques and Dapi (blue) was used as nucleus stain. One-way ANOVA with Tukey’s Multiple Comparison Test (A, B, F *n* = 3/group) was performed. Scale: 50 μm (C, D, E), 20 μm (G). (TIF 4515 kb)

Additional file 7: **Figure S7.** Flow cytometric data of brain isolated T-cells were analyzed for gender-specific differences: (A) Female APP-PS1 mice had significantly increased numbers of CD3^+^ T-cells when compared specifically to female WT mice, however this was not seen in male mice (B). (C) Female APP-PS1 had increased numbers of CD3^+^ T-cells compared to male APP-PS1 mice. (D) The number of CD3^+^/CD4^+^ T-cells was significantly increased in female APP-PS1 compared to female WT mice, whereas male mice of both genotypes had the same cell numbers in the brain (E). (F) APP-PS1 female mice had higher numbers of CD3^+^/CD4^+^ T-cells compared to male APP-PS1 mice. (G) There was a trend for higher CD3^+^/CD8^+^ T-cell numbers in the brain of female APP-PS1 mice compared to female WT mice, however this was not seen in male animals (H). (I) Female APP-PS1 mice had higher numbers of CD3^+^/CD8^+^ T-cells compared to male APP-PS1 animals. PLX5622 treatment in APP-PS1 mice of both sexes showed an increase in the number of CD3^+^, CD3^+^/CD4^+^ and CD3^+^/CD8^+^ T-cells in the brain. One-way ANOVA with Tukey’s Multiple Comparison Test (A, B, D, E, G, H, n = 3–5/group) and Unpaired Student’s T-test (A, C, D, F, G, I) for comparison of only two groups were performed (n = 3–5/group). (TIF 743 kb)

Additional file 8: **Figure S8.** Immunohistochemical staining for proliferating cell nuclear antigen (PCNA) to analyze the proliferative activity of CD8^+^ T-cells in APP-PS1 and microglia ablated APP-PS1 brains. CD8^+^ T-cells in the hippocampus (A) and cortex (B) of APP-PS1 mice were not observed to proliferate (arrows). Interestingly, after microglia ablation in APP-PS1 mice, no increase in proliferative activity was detected, suggesting CD8^+^ T-cells to rather infiltrate the brain than being locally generated. Dapi (blue) was used as nucleus stain. Scale: 50 μm (A, B). (TIF 4664 kb)

Additional file 9: **Figure S9.** Immunohistochemical analysis for CD4^+^ T-cells and Zap70 expression in APP-PS1 mouse brains. CD4^+^ T-cells were observed less frequently than CD8^+^ T-cells in hippocampal (A) and cortical (B) brain regions and expressed the T-cell receptor kinase Zap70 (arrow). Dapi (blue) was used as nucleus stain. Scale: 50 μm (A, B). (TIF 2033 kb)

## Abbreviations

AD: Alzheimer’s disease
AIF1: Allograft inflammatory factor 1
ALS: Amyotrophic lateral sclerosis
APC: Antigen presenting cell
APP-PS1: APP Swedish PS1 dE9
Arg1: Arginase 1
CCL2: Chemokine (C-C motif) ligand 2 or monocyte chemoattractant protein 1
CD11b: Integrin alpha M
CD3: Cluster of differentiation 3, T-cell co-receptor
CD33: Cluster of differentiation 33, sialic acid binding Ig-like lectin 3
CD4: Cluster of differentiation 4, transmembrane glycoprotein
CD44: Cluster of differentiation 44, receptor for hyaluronic acid
CD45: Cluster of differentiation 45, protein tyrosine phosphatase receptor type C
CD68: Cluster of differentiation 68
CD8: Cluster of differentiation 8, transmembrane glycoprotein
CNS: Central nervous system
CSF1R: Colony stimulating factor 1 receptor
H2-Aa: Histocompatibility 2, class II antigen A, alpha
HA: Hyaluronan
HSVTK: Herpes simplex virus thymidine kinase
Iba1: Ionized calcium-binding adapter molecule 1
IL10: Interleukin 10
IL-1beta: Interleukin 1 beta
IL6: Interleukin 6
INF: Interferon
Marco: Macrophage receptor with collagenous structure
MHCII: Major histocompatibility complex 2
MRC1: Mannose receptor C-Type 1
MS: Multiple sclerosis
MWM: Morris water maze
Nos2: Nitric oxide synthase 2
PD: Parkinson’s disease
TCR: T-cell receptor
TGFbeta: Transforming growth factor beta
TMEM119: Transmembrane protein 119
TNF: Tumor necrosis factor
Treg: Regulatory T-cells
Trem2: Triggering receptor expressed on myeloid cells 2
WT: Wild type
