# Supplementary figures and images for: Microglia prevent peripheral immune cell invasion and promote an anti-inflammatory environment in the brain of APP-PS1 transgenic mice
Source: J Neuroinflammation. 2018 Sep 21;15:274. doi: 10.1186/s12974-018-1304-4 (PMC6151006; doi:10.1186/s12974-018-1304-4)

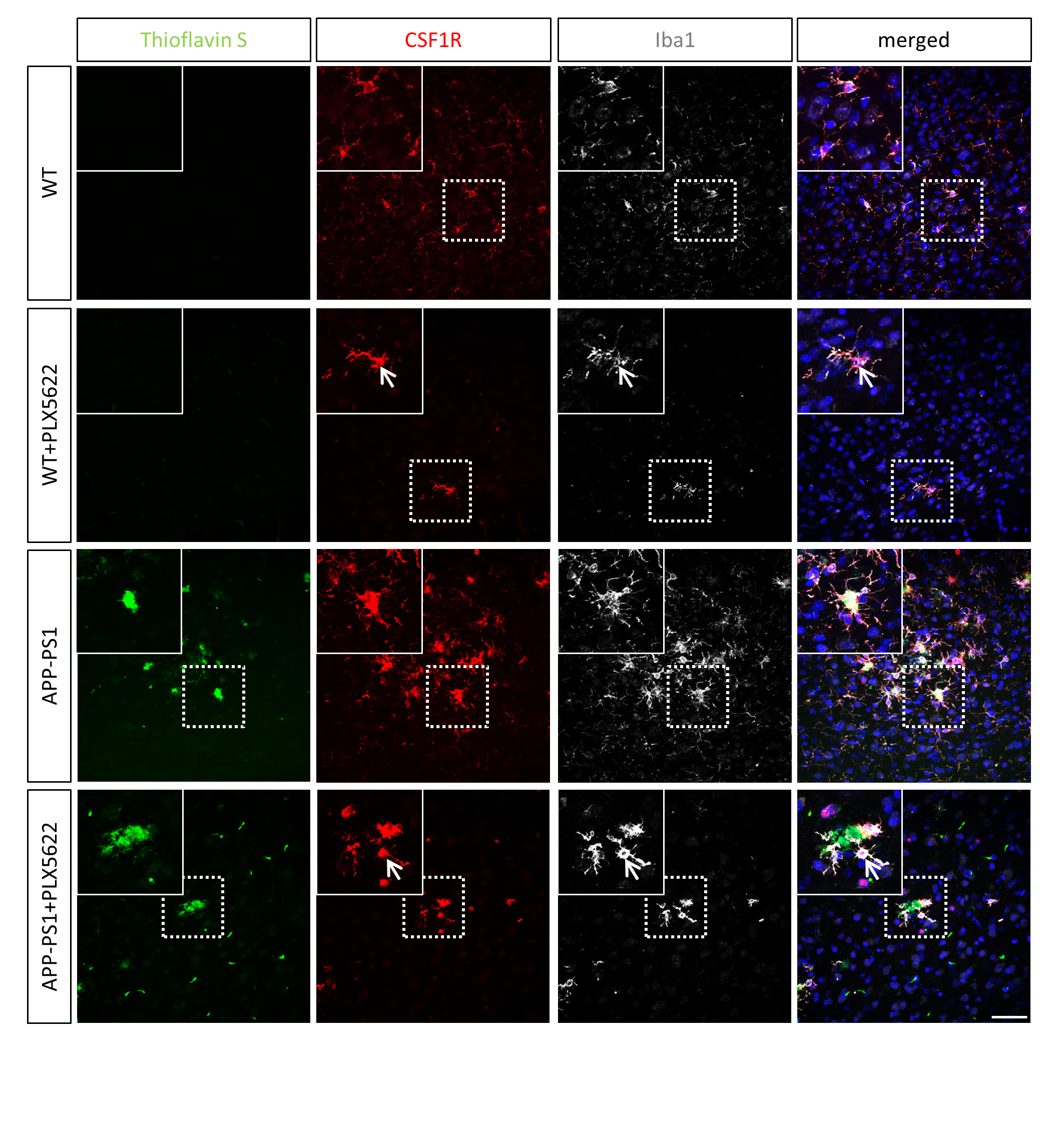

Supplement: Supplementary file 1 — Figure S1. Qualitative immunohistochemical staining for CSF1R receptor (red) in mouse brain cortex showed high expression in Iba1+ cells (white) in all studied groups. ThioflavinS was used to stain amyloid plaques (green) and Dapi (blue) was used as nucleus stain. Scale: 50 μm. (TIF 3837 kb) [file 12974_2018_1304_MOESM1_ESM.tif]

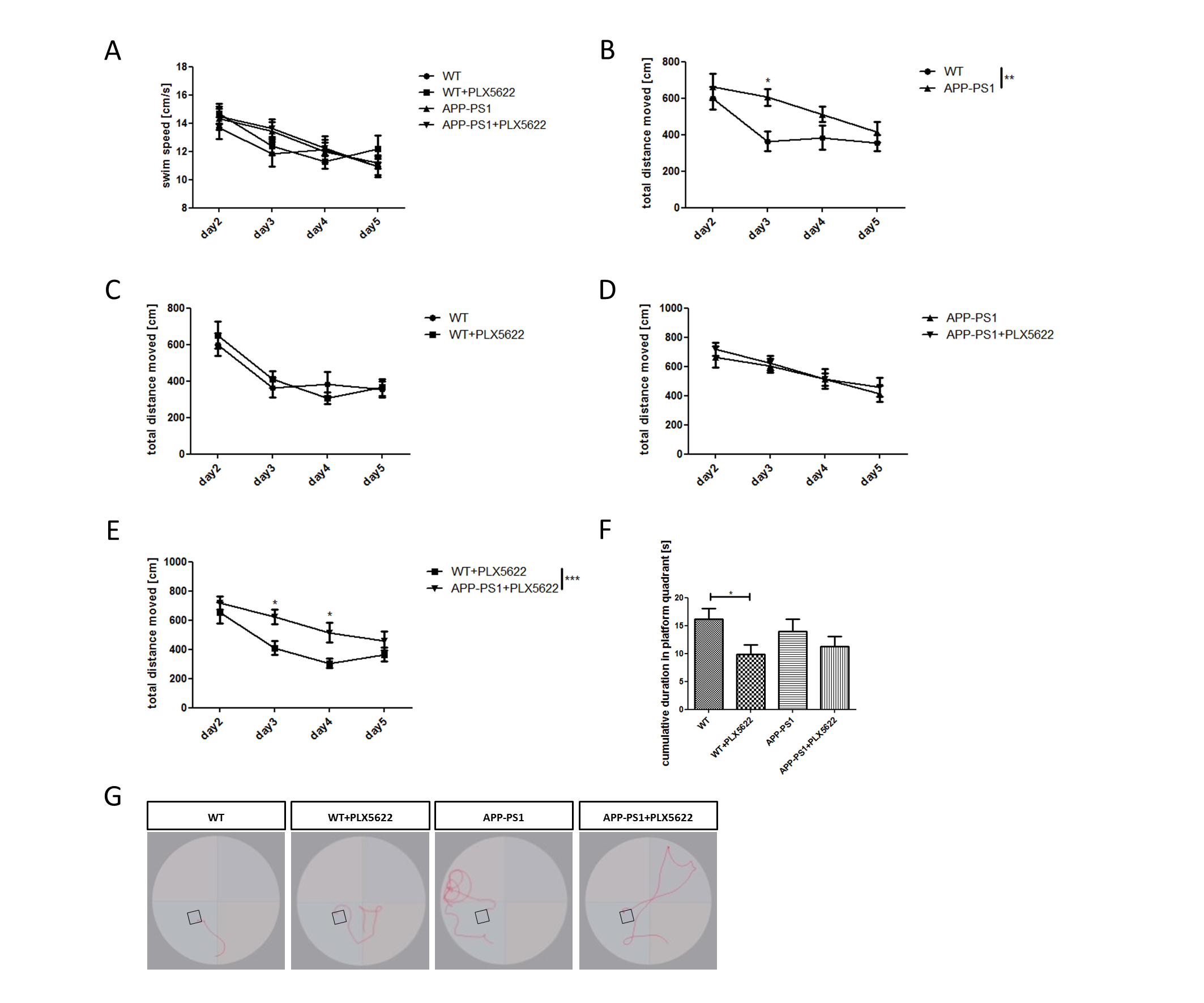

Supplement: Supplementary file 2 — Figure S2. Microglia ablation has no impact on learning behavior and did not improve learning deficits in APP-PS1 animals. Morris Water Maze (MWM) test for spatial learning and memory was performed and the total distance the animals moved to reach the platform was calculated as measure for learning improvement. (A) All animals moved with the same swim speed. There was a significant difference between the total distances traveled to reach the platform from day 2 to day 5 in APP-PS1 mice compared to WT animals (B). In WT mice PLX5622 treatment has no impact on the total distance the animals moved to reach the platform (C). Microglia ablation in APP-PS1 mice did not improve learning deficits compared to untreated APP-PS1 mice (D) or WT PLX5622 treated mice (E). Spatial memory was tested on day 6 after platform removal and the duration of the animals in the original platform quadrant was measured (F). There was no significant differences comparing all 4 groups, however WT PLX5622 treated mice spent decreased time in the original platform quadrant when only compared to WT mice (F). Representative track visualization of the total distances traveled at day 5 in MWM test (G). Data are shown as mean with SEM (A-F). Two-way ANOVA with Bonferroni Post-test was performed (A-E, n = 9/group) and One-way ANOVA with Tukey’s Multiple Comparison test or Unpaired Student’s T-test were performed comparing only WT with WT + PLX5622 (F, n = 9/group). (TIF 536 kb) [file 12974_2018_1304_MOESM2_ESM.tif]

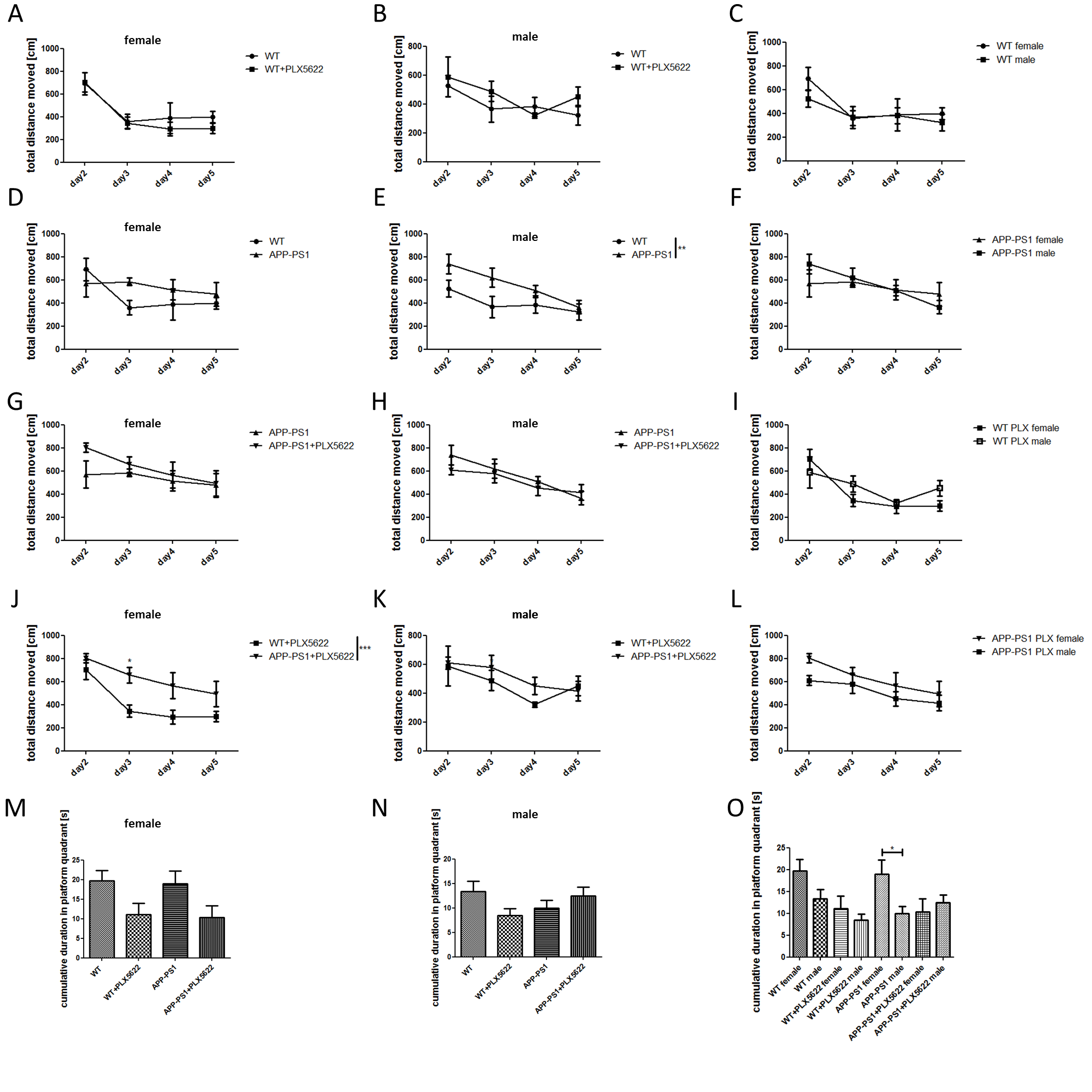

Supplement: Supplementary file 3 — Figure S3. Behavioral data of the Morris Water Maze test were analyzed for gender specific differences: Microglia ablation had no sex-specific impact on learning behavior in female (A) or male (B) WT mice and there was no gender-specific difference in the total distance WT mice moved to reach the platform (C). APP-PS1 female (D) and male (E) mice traveled higher distances to reach the platform compared to female and male WT mice, however no gender differences were observed in APP-PS1 mice (F). Microglia ablation in APP-PS1 mice did not improve learning deficits in female or male mice compared to either untreated APP-PS1 mice (G, H) or WT PLX5622 treated mice of corresponding gender (J, K). There was no difference in the distance moved between female and male WT mice treated with PLX5622 (I). Female and male APP-PS1 microglia ablated mice showed no sex difference in the distance moved to reach the platform (L). Spatial memory was tested on day 6 after platform removal and the duration of the animals in the original platform quadrant was measured. A trend for reduced memory of the spatial platform location was observed in female WT PLX5622 and female APP-PS1 PLX5622 treated mice compared to respective controls (M), but no significant difference was observed in male PLX5622 treated animals (N). Comparison of the duration in the platform quadrant in female versus male mice for the single studied groups showed a significant reduction in spatial memory in male APP-PS1 mice compared to female APP-PS1 (O). Data are shown as mean with SEM (A-O). Two-way ANOVA with Bonferroni Post-test (A-L, n = 4–5/group), One-way ANOVA with Tukey’s Multiple Comparison test (M, N, n = 4–5/group) and Unpaired Student’s T-test for comparison of female versus male in the respective groups (O, n = 4–5/group) were performed. (TIF 867 kb) [file 12974_2018_1304_MOESM3_ESM.tif]

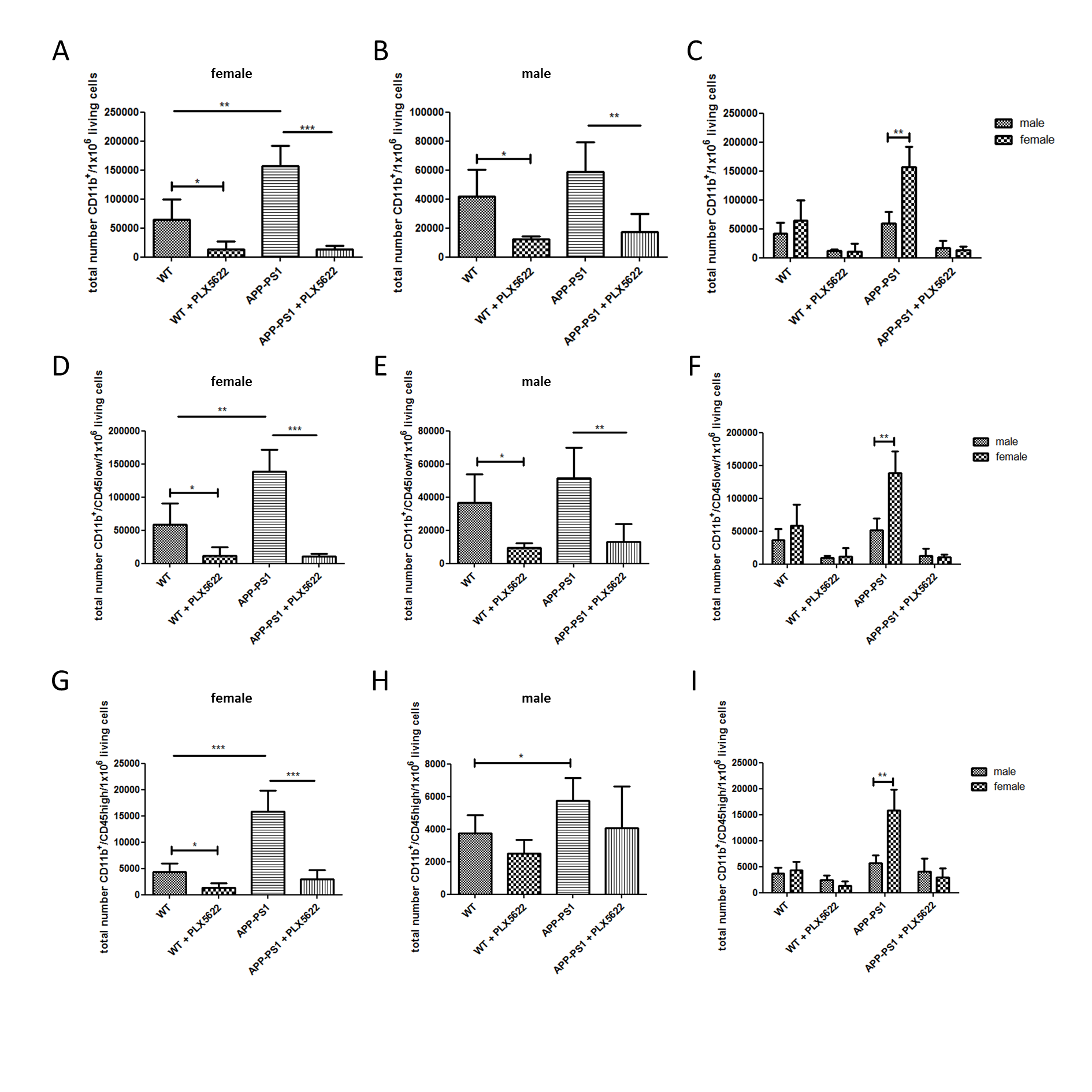

Supplement: Supplementary file 4 — Figure S4. Detailed analysis for gender-specific differences of flow cytometric data from brain isolated microglia/macrophage populations: PLX5622 treatment reduced CD11b+ cell numbers in the brain of female (A) and male (B) animals, however specifically female APP-PS1 mice had higher numbers of CD11b+ cells compared to female WT mice. Gender-specific differences were observed in APP-PS1 mice, where the females had higher numbers of CD11b+ cells compared to male APP-PS1 mice (C). Similar results were obtained from microglia cell numbers (CD11b+/CD45low) of APP-PS1 mice with increased numbers of microglia in female APP-PS1 compared to female WT mice and compared to male APP-PS1 animals (D-F). Macrophage numbers (CD11b+/CD45high) where significantly increased in female APP-PS1 compared to female WT mice and were reduced upon PLX5622 treatment in both genotypes (G). Also male APP-PS1 mice had increased numbers of CD11b+/CD45high cells compared to male WT mice, however PLX5622 treatment did not reduce macrophage numbers in male animals of both genotypes (H). Higher numbers of macrophages were already detected in the brains of female APP-PS1 mice compared to male APP-PS1 animals (I). One-way ANOVA with Tukey’s Multiple Comparison test (A, B, D, E, G, H n = 4–5/group) and Unpaired Student’s T-test (A, C, D, F, G, H, I) with Welch’s correction (B, E) for comparison of only two groups were performed (n = 4–5/group). (TIF 727 kb) [file 12974_2018_1304_MOESM4_ESM.tif]

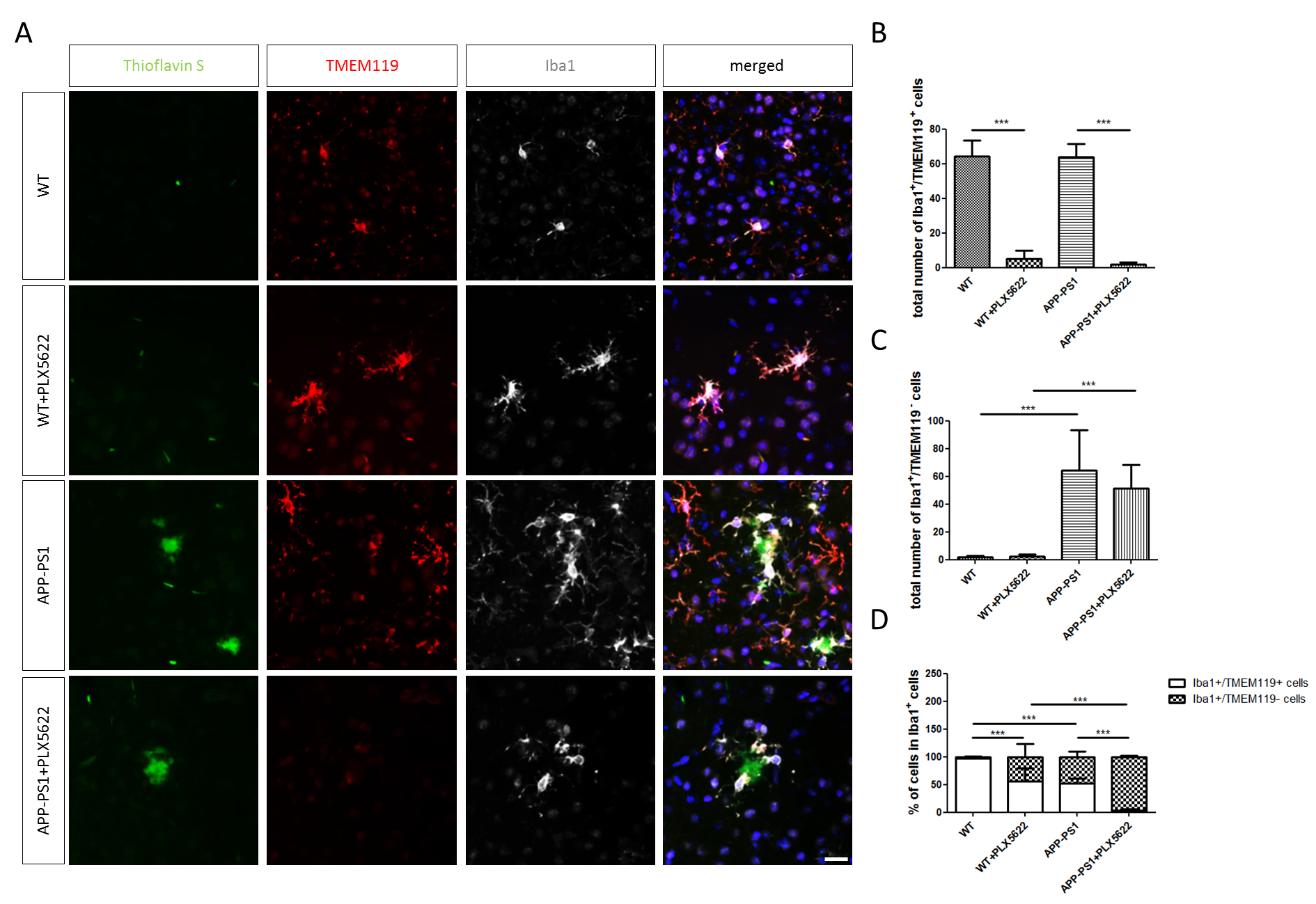

Supplement: Supplementary file 5 — Figure S5. Using the newly identified microglia specific marker TMEM119 for detailed immunohistochemical analysis in the cortex revealed strong co-localization of Iba1+ (white) cells with TMEM119 (red) in WT and WT animals treated with PLX5622 (A). However, Iba1+ cells at sites of plaques (green) in APP-PS1 and PLX5622 treated APP-PS1 animals did not express TMEM119. Quantitative analysis of Iba1+/TMEM119+ revealed a significant reduction in Iba1+/TMEM119+ cell numbers upon PLX5622 treatment in WT and APP-PS1 mice (B). Surprisingly, APP-PS1 animals had increased numbers of Iba1+/TMEM119− cells that were more resistant to PLX5622 treatment than in WT animals (C). Calculation of the percentage of Iba1+/TMEM119+ and Iba1+/TMEM119− cells from the total Iba1+ cell population (D). ThioflavinS was used to stain amyloid plaques (green) and Dapi (blue) was used as nucleus stain. One-way ANOVA with Tukey’s Multiple Comparison Test (B, C, D n = 6/group) was performed. Scale: 20 μm (A). (TIF 1310 kb) [file 12974_2018_1304_MOESM5_ESM.tif]

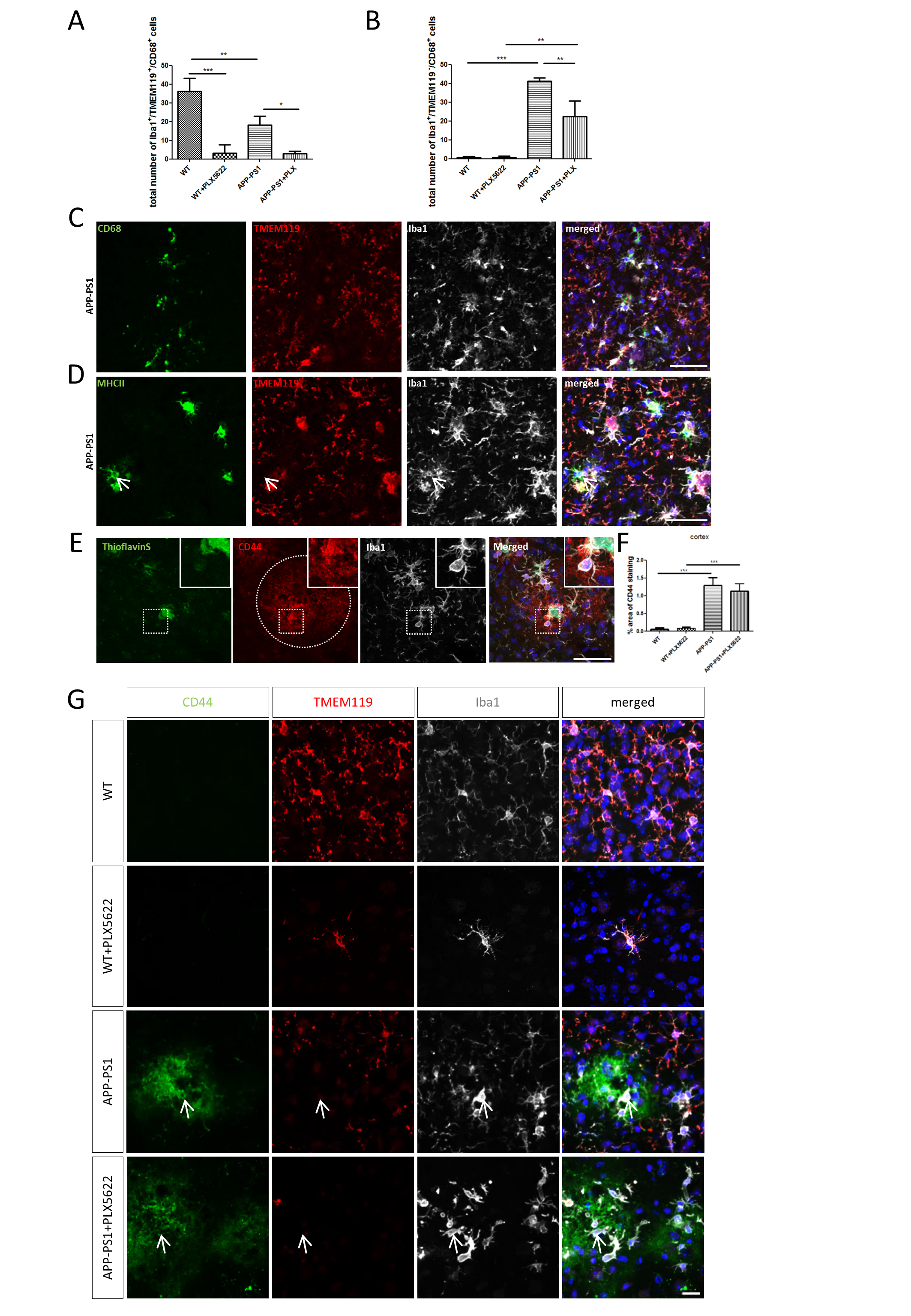

Supplement: Supplementary file 6 — Figure S6. Iba1+/TMEM119− cells represent a CD68+ macrophage population with peripheral origin highly involved in amyloid-beta phagocytosis. Analysis of CD68 expression in the cortex revealed significantly reduced numbers of Iba1+/TMEM119+/CD68+ cells in APP-PS1 and WT animals upon PLX5622 treatment (A). Surprisingly, higher numbers of Iba1+/TMEM119−/CD68+ cells were found in APP-PS1 animals compared to WT, although these numbers were slightly reduced in APP-PS1 animals by PLX5622 treatment (B). Representative image of CD68 expression in Iba1+/TMEM119− cells located at sites of plaque in APP-PS1 mice (C). Strong MHCII expression was seen sporadically in Iba1+/TMEM119− cells in APP-PS1 mice (D, arrow). Accumulation of CD44 staining (red) was observed extracellularly around amyloid depositions (green) as indicated by the doted ellipse and CD44 staining was seen on Iba1+ cells at sites of plaques (E, insert). Quantification of percentage (%) area of CD44 staining in hippocampal brain regions revealed barely any staining in WT and WT + PLX5622 treated animals, however in APP-PS1 and APP-PS1 + PLX5622 treated mice significantly higher expression of CD44 was observed compared to WT or WT + PLX5622 animals (F). Detailed immunohistochemical analysis revealed increased staining for CD44 at sites of amyloid deposition in areas colonized with Iba1+/TMEM119− cells in APP-PS1 and APP-PS1 PLX5622 treated mice (G, arrow). ThioflavinS was used to stain amyloid plaques and Dapi (blue) was used as nucleus stain. One-way ANOVA with Tukey’s Multiple Comparison Test (A, B, F n = 3/group) was performed. Scale: 50 μm (C, D, E), 20 μm (G). (TIF 4515 kb) [file 12974_2018_1304_MOESM6_ESM.tif]

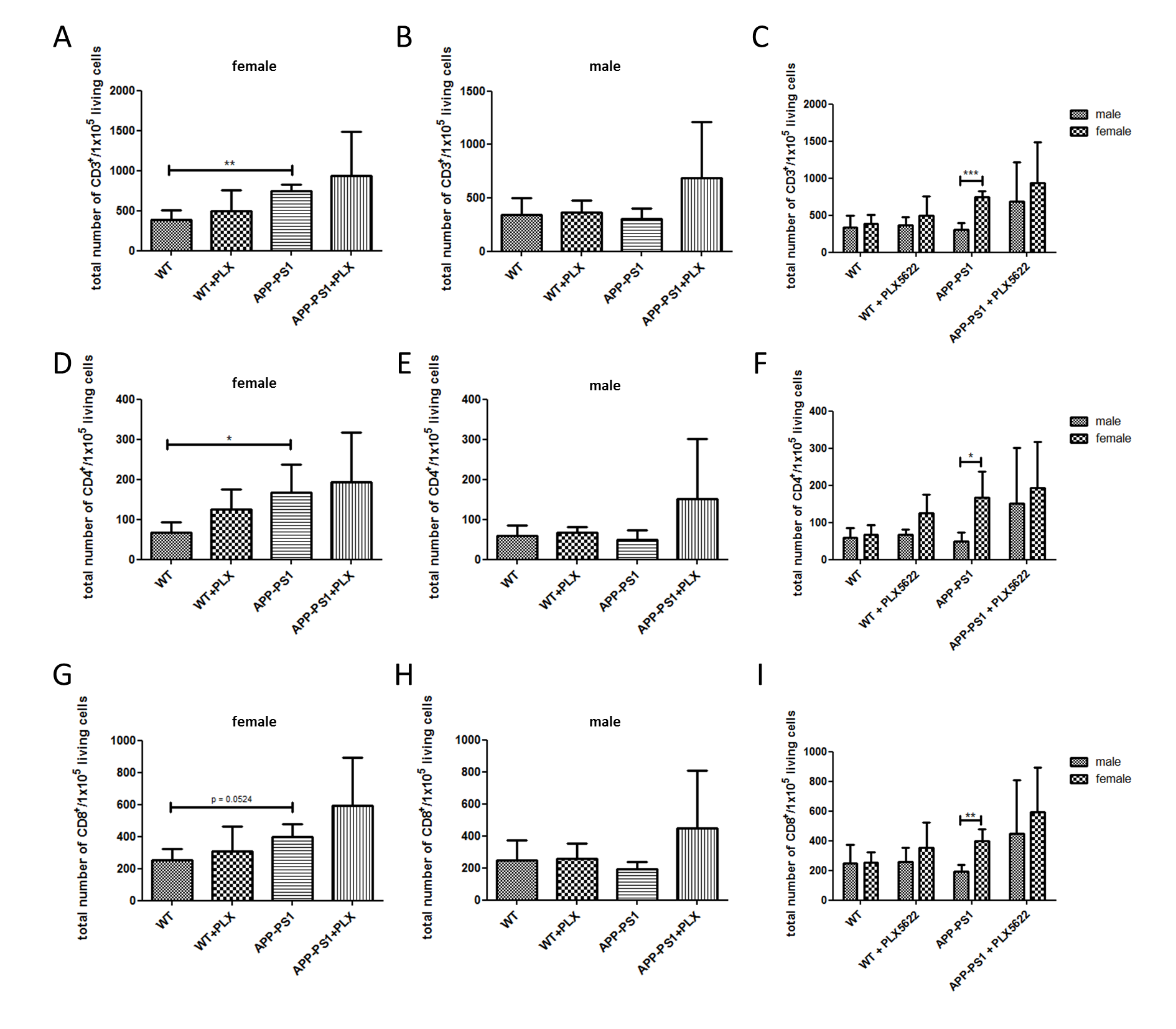

Supplement: Supplementary file 7 — Figure S7. Flow cytometric data of brain isolated T-cells were analyzed for gender-specific differences: (A) Female APP-PS1 mice had significantly increased numbers of CD3+ T-cells when compared specifically to female WT mice, however this was not seen in male mice (B). (C) Female APP-PS1 had increased numbers of CD3+ T-cells compared to male APP-PS1 mice. (D) The number of CD3+/CD4+ T-cells was significantly increased in female APP-PS1 compared to female WT mice, whereas male mice of both genotypes had the same cell numbers in the brain (E). (F) APP-PS1 female mice had higher numbers of CD3+/CD4+ T-cells compared to male APP-PS1 mice. (G) There was a trend for higher CD3+/CD8+ T-cell numbers in the brain of female APP-PS1 mice compared to female WT mice, however this was not seen in male animals (H). (I) Female APP-PS1 mice had higher numbers of CD3+/CD8+ T-cells compared to male APP-PS1 animals. PLX5622 treatment in APP-PS1 mice of both sexes showed an increase in the number of CD3+, CD3+/CD4+ and CD3+/CD8+ T-cells in the brain. One-way ANOVA with Tukey’s Multiple Comparison Test (A, B, D, E, G, H, n = 3–5/group) and Unpaired Student’s T-test (A, C, D, F, G, I) for comparison of only two groups were performed (n = 3–5/group). (TIF 743 kb) [file 12974_2018_1304_MOESM7_ESM.tif]

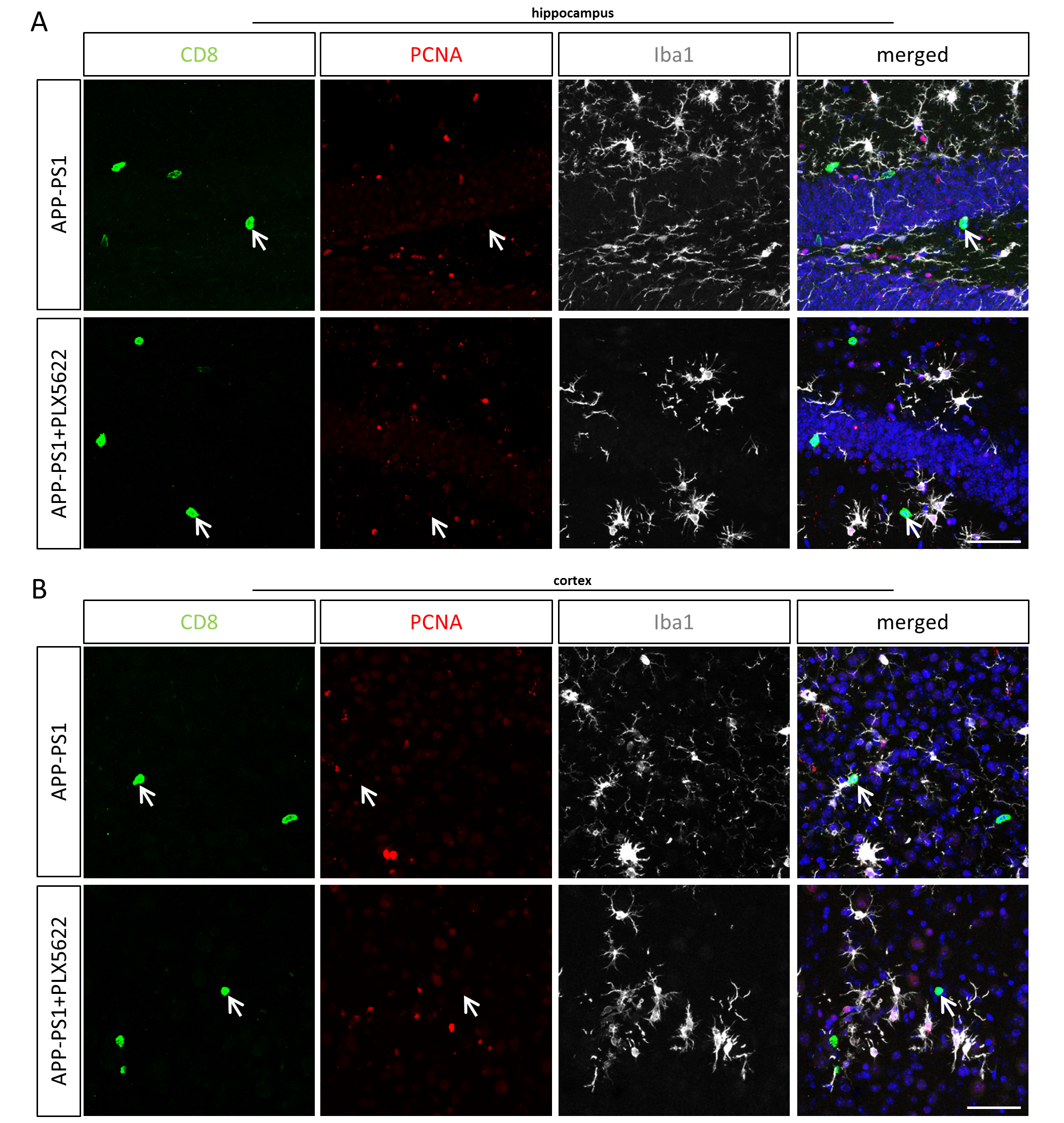

Supplement: Supplementary file 8 — Figure S8. Immunohistochemical staining for proliferating cell nuclear antigen (PCNA) to analyze the proliferative activity of CD8+ T-cells in APP-PS1 and microglia ablated APP-PS1 brains. CD8+ T-cells in the hippocampus (A) and cortex (B) of APP-PS1 mice were not observed to proliferate (arrows). Interestingly, after microglia ablation in APP-PS1 mice, no increase in proliferative activity was detected, suggesting CD8+ T-cells to rather infiltrate the brain than being locally generated. Dapi (blue) was used as nucleus stain. Scale: 50 μm (A, B). (TIF 4664 kb) [file 12974_2018_1304_MOESM8_ESM.tif]

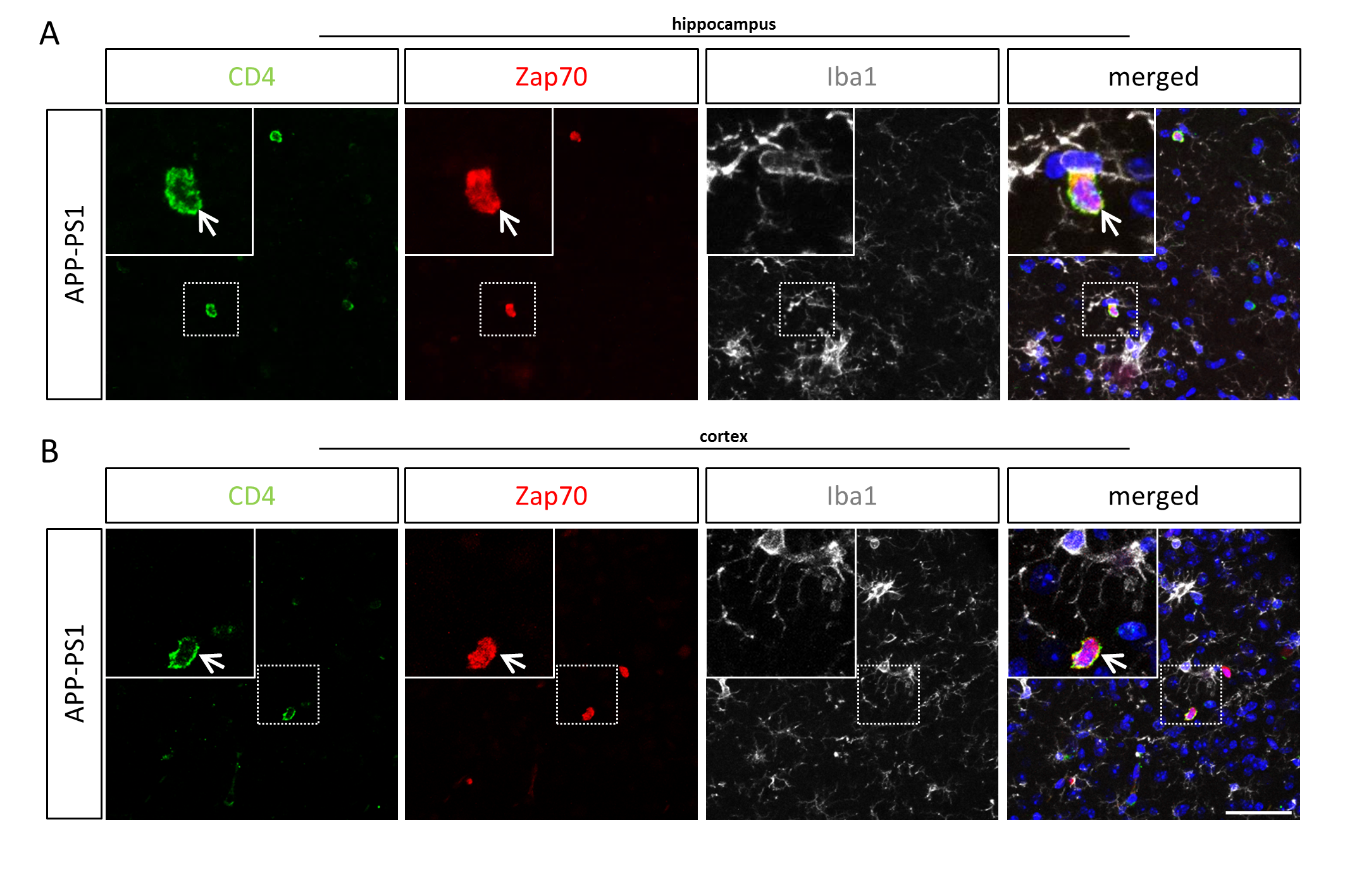

Supplement: Supplementary file 9 — Figure S9. Immunohistochemical analysis for CD4+ T-cells and Zap70 expression in APP-PS1 mouse brains. CD4+ T-cells were observed less frequently than CD8+ T-cells in hippocampal (A) and cortical (B) brain regions and expressed the T-cell receptor kinase Zap70 (arrow). Dapi (blue) was used as nucleus stain. Scale: 50 μm (A, B). (TIF 2033 kb) [file 12974_2018_1304_MOESM9_ESM.tif]
